# RNA Helicase DDX5 in Association With IFI16 and the Polycomb Repressive Complex 2 Silences Transcription of the Hepatitis B Virus by Interferon

**DOI:** 10.1002/jmv.70118

**Published:** 2024-12-16

**Authors:** Zhili Li, Naimur Rahman, Cheng Bi, Rodrigo Mohallem, Aryamav Pattnaik, Majid Kazemian, Fang Huang, Uma K. Aryal, Ourania Andrisani

**Affiliations:** ^1^ Department of Basic Medical Sciences Purdue University West Lafayette Indiana USA; ^2^ Purdue Institute for Cancer Research Purdue University West Lafayette Indiana USA; ^3^ Department of Biomedical Engineering Purdue University West Lafayette Indiana USA; ^4^ Purdue Proteomics Facility, Bindley Bioscience Center Purdue University West Lafayette Indiana USA; ^5^ Department of Comparative Pathobiology Purdue University West Lafayette Indiana USA; ^6^ Department of Biochemistry Purdue University West Lafayette Indiana USA; ^7^ Department of Computer Science Purdue University West Lafayette Indiana USA

**Keywords:** interferon Inducible protein 16, polycomb repressive complex 2, pyrin and hematopoietic interferon‐inducible nuclear (HIN) domain (PYHIN) family, RNA helicase DEAD box protein 5, single‐molecule localization microscopy (SMLM)

## Abstract

RNA helicase DDX5 is a host restriction factor for hepatitis B virus (HBV) biosynthesis. Mass spectrometry (LC‐MS/MS) identified significant DDX5‐interacting partners, including interferon‐inducible protein 16 (IFI16) and RBBP4/7, an auxiliary subunit of polycomb repressive complex 2 (PRC2). DDX5 co‐eluted with IFI16, RBBP4/7, and core PRC2 subunits in size exclusion chromatography fractions derived from native nuclear extracts. Native gel electrophoresis of DDX5 immunoprecipitants revealed a 750 kDa DDX5/IFI16/PRC2 complex, validated by nanoscale co‐localization via super‐resolution microscopy. Prior studies demonstrated that IFI16 suppresses HBV transcription by binding to the interferon‐sensitive response element of covalently closed circular DNA (cccDNA), reducing H3 acetylation and increasing H3K27me3 levels by an unknown mechanism. Herein, we demonstrate that ectopic expression of IFI16 inhibited HBV transcription from recombinant rcccDNA, correlating with increased IFI16 binding to rcccDNA, reduced H3 acetylation, and elevated H3K27me3, determined by chromatin immunoprecipitation. Importantly, the inhibitory effect of ectopic IFI16 on HBV transcription was reversed by siRNA‐mediated knockdown of DDX5 and EZH2, the methyltransferase subunit of PRC2. This reversal was associated with decreased IFI16 binding to rcccDNA, enhanced H3 acetylation, and reduced H3K27me3. Similarly, endogenous IFI16 induced by interferon‐α inhibited HBV rcccDNA transcription in a DDX5‐ and PRC2‐dependent manner. In HBV‐infected HepG2‐NTCP cells, the antiviral effect of interferon‐α was abrogated upon knockdown of DDX5 and EZH2, underscoring the crucial role of the DDX5 complex in IFI16‐mediated antiviral response. In conclusion, in response to interferon, DDX5 partners with IFI16 to bind cccDNA, directing PRC2 to epigenetically silence cccDNA chromatin, thereby regulating immune signaling and HBV transcription.

## 1 Introduction

Chronic infection by the hepatitis B virus (HBV) is a major etiologic factor in hepatocellular carcinoma (HCC) pathogenesis [1]. A high level of viremia in chronic HBV patients is a prognostic indicator that the disease will advance to HCC [2]. Despite the availability of the HBV vaccine, the World Health Organization (WHO) estimates that 250 million are chronic HBV carriers globally, having an increased risk for developing liver cancer, which is usually fatal. Curative treatments (nucleos(t)ide analogs and pegylated interferon‐α) for chronic HBV infection and HBV‐related HCC remain ineffective [3] due to viral persistence [4]. Despite the use of these potent antivirals, a functional cure for HBV infection is rarely achieved following interferon therapy [3]. Further studies are required for developing alternative strategies to silence viral transcription, and potentially achieve a cure for chronic HBV infection.

The mechanism of HBV biosynthesis [5] involves the synthesis of the covalently closed circular DNA (cccDNA), forming a chromatin‐like structure in the host cell nucleus known as the HBV mini‐chromosome. In its active state, the cccDNA chromatin serves as template for all viral transcripts including pregenomic RNA (pgRNA), which acts as template for synthesis of the viral genome [5]. The level of HBV replication and viremia of HBV‐infected patients correlates with the acetylation status of H3 and H4 of cccDNA chromatin [6]. Additionally, prolonged treatment of patients with the nucleoside analog telbivudine increases the level of H3K27me3, a repressive histone modification associated with transcriptional silencing of the HBV mini‐chromosome [7].

These findings imply that the recruitment of specific epigenetic complexes to the HBV mini‐chromosome is responsible for either the transcription‐activating or silencing histone modifications on the viral chromatin. Ultimately, these modifications determine the rate of HBV transcription and replication. However, the precise mechanism(s) underlying the recruitment of specific epigenetic complexes to the cccDNA chromatin for mediating the activating or repressive histone modifications remain to be determined.

Our previous studies demonstrated that downregulation of the polycomb repressive complex 2 (PRC2) in HBV‐infected cells [8] led to increased transcription of viral genes and specific cellular genes associated with poor HCC prognosis [9, 10]. In addition, we identified DDX5 as an important regulator of PRC2. Specifically, through interaction with SUZ12, a core PRC2 subunit, DDX5 stabilizes PRC2 and inhibits HBV biosynthesis [11]. DDX5 is a DEAD box RNA helicase [12, 13] involved in various aspects of RNA biology in vivo, functioning by hydrolyzing ATP and mediating ATP‐dependent RNA binding and remodeling [14]. One significant function of DDX5 in the context of HBV infection is regulation of STAT1 translation, by resolving a G‐quadruplex located at the 5′ UTR of STAT1 mRNA [15]. Since STAT1 is a key transcription factor in interferon signaling, this result suggests a role of DDX5 in immune signaling pathways and viral gene expression [15].

In this study, we performed label‐free quantitative proteomic analysis using LC‐MS/MS to identify cellular interacting partners of DDX5. We identified interferon‐inducible protein 16 (IFI16), as the most prominent cellular protein interacting with DDX5. IFI16 belongs to the pyrin and HIN‐200 family of proteins, known as PYHIN, containing two HIN‐200‐aa DNA binding domains at the C‐terminus, and a pyrin domain at the N‐terminus [16].

IFI16 is located primarily in the nucleus, where it exerts antiviral effects by repressing viral transcription rather than through viral DNA sensing [16]. IFI16 targets various viruses operating in the nucleus, employing diverse mechanisms of antiviral action. Specifically, IFI16 inhibits human cytomegalovirus transcription [17], restricts HSV‐1 replication and gene expression by modulating histone modifications [18], represses human papilloma virus gene expression [19], and inhibits HIV‐1 transcription by inhibiting the transcription factor SP1 [20]. IFI16, also, epigenetically suppresses transcription from the HBV cccDNA [21], although the precise mechanism by which IFI16 achieves this epigenetic silencing of HBV transcription remains unknown.

In this study, we demonstrate that DDX5 forms an RNA‐based epigenetic silencing complex comprised of IFI16 and PRC2 subunits. We found that expression of ectopic IFI16 or endogenous IFI16 induced by IFN‐α, represses transcription from cccDNA of HBV [22]. Importantly, the transcription silencing effect of IFI16 on rcccDNA [22] is abrogated by downregulation of DDX5, RBBP4, EZH2, or SUZ12 leading to restoration of HBV infection.

Given that IFI16 is an IFN‐α stimulated gene [21], our findings suggest that the transcription‐silencing effect of IFN‐α is dependent on both IFI16 [21] and DDX5, mediated by the interacting PRC2 complex. Thus, the host‐restriction function of DDX5 involves the assembly of an RNA‐driven multicomponent complex comprised of IFI16 and PRC2, which orchestrates histone modifications silencing the HBV mini‐chromosome. Considering that DDX5 regulates STAT1 translation [15] and that IFN‐α induces IFI16 via STAT1 [23, 24], we conclude that DDX5 exerts a key role in immune signaling pathways that regulate HBV transcription. We propose that the absence of key components of this DDX5‐based epigenetic silencing complex contributes to HBV persistence. Intriguingly, IFI16 levels are reduced in the liver tissue of chronically HBV‐infected patients [21].

## 2 Materials and Methods

### 2.1 Cell Culture and Transfections

HepaRG, Huh7, and HepG2‐NTCP cells [25] were grown in DMEM/F12 supplemented with 10% FBS. Transfections of recombinant circular covalently closed HBV rcccDNA were performed as previously described [22].

### 2.2 Chromatin Immunoprecipitation (ChIP) Assays

ChIP assays were performed using the SimpleChIP Enzymatic Chromatin IP Kit (Magnetic Beads) (Cell Signaling Technology). HepG2‐NTCP cells were transfected with prcccDNA/pCRE plasmids and siRNAs for 72 h, followed by cross‐linking with formaldehyde to a final concentration of 1%. Approximately 4 × 10^6^ cells were used per ChIP assay. Immunoprecipitations were carried out with the indicated antibodies (Supporting Information S1: Table S2). After reversing the cross‐links, DNA was extracted and subjected to real‐time quantitative PCR (qPCR) using p1 and p2 primers (Supporting Information S1: Table S3).

### 2.3 HBV Infections

Virus preparation was performed using HepDE19 cells [26], and the infection of HepG2‐NTCP cells was conducted as previously described [26, 27]. Notably, 4% polyethylene glycol (PEG) was included in the infected cell media only on the first day of infection. Detailed protocol for HBV infection and flow cytometry were as described earlier [26], included in Supporting Information. Quantification of cccDNA in HBV infected cells was performed using Hirt extraction of viral DNA, followed by T5 exonuclease reaction and PCR amplification as described [27].

Nuclear extract preparation, size exclusion chromatography (SEC), proteomics sample preparation, liquid chromatography‐tandem mass spectrometry (LC‐MS/MS) analysis and mass spectrometry data analysis are described in detailed protocols, included in Supporting Information.

Immunoblots, siRNA transfections, and qRT‐PCR were performed as previously described [15, 28]. For quantification of HBV RNAs, PrimeScript Reagent Kit with gDNA Eraser was used to remove contaminating transfected HBV DNA [21]. Supporting Information S1: Tables S1–S4 list plasmids, siRNA sequences, antibodies, primer sequences for qRT‐PCR, reagents, chemicals, and kits.

### 2.4 Native Polyacrylamide Gel Electrophoresis

Native nuclear lysates prepared from three 10 cm plates of doxycycline (Dox)‐inducible HepaRG cells expressing FLAG‐DDX5 (treated with 1.0 µg/mL Dox and 500 ng/mL IFN‐α for 48 h) were immunoprecipitated with anti‐DDX5 overnight at 4°C, followed by overnight incubation at 4°C with 40 µL protein A/G magnetic beads. Immunoprecipitants were eluted twice with 40 µL of 0.2 M glycine pH 2.6 and neutralized by addition of equal volume of 0.1 M Tris‐HCL pH 8.0. Samples were electrophoresed using NativePAGE 3%–12%, Bis‐Tris, 1.0 mm Miniprotein Gels (Thermofisher), with NativePAGE Running Buffer (Thermofisher) and NativeMark unstained protein standard (Thermofisher), transferred to PVDF membrane and subjected to immunoblot analysis.

### 2.5 Super Resolution Two‐Color Microscopy

Two‐color super‐resolution data were collected using a custom‐designed single‐molecule localization microscopy (SMLM) setup with an Olympus IX‐73 microscope stand. Detailed protocols for cell fixation, imaging buffers, sample mounting, microscope set‐up, and SMLM acquisition and reconstruction are described in Supporting Information.

### 2.6 Quantification of Co‐Localization

SMLM generates molecular coordinates in two‐ or three‐dimensional cellular space with a resolution of 10–20 nm. Quantification of the co‐localization of two proteins at different length scales was performed by Voronoï tessellation‐based relative enrichment (RE) quantification between two fluorescent targets [29]. The approach calculates the RE of one molecular species based on the density distribution of the second reference species, shorted and plotted by the mean nearest neighbor distance of the reference polygons from Voronoï tessellation. For two uncorrelated molecular species, the RE ≈ 1, whereas for spatially correlated species RE > 1. Detailed information for RE quantification and correlated simulation method is described in Supporting Information and Supporting Information S1: Figures S1–S5.

### 2.7 Statistical Analysis

Statistical analysis performed using unpaired *t* test in GraphPad Prism version 6.0 (GraphPad Software, San Diego, CA). Differences were considered significant when *p* < 0.05.

## 3 Results

**Figure 1:**
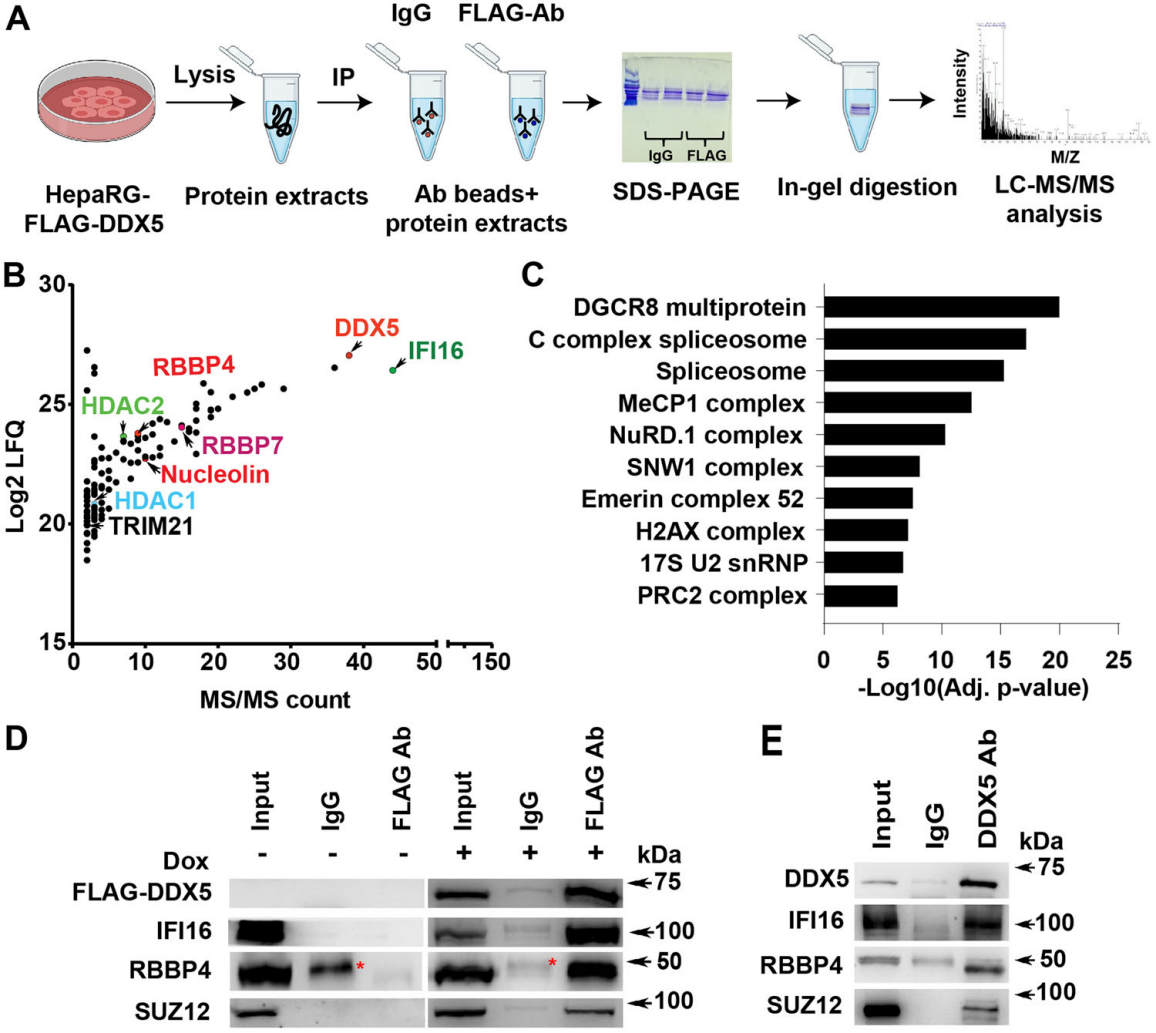
IFI16 is a major DDX5 interacting protein. (A) Schematic of experimental workflow. Doxycycline inducible FLAG‐DDX5 expressing HepaRG cells, grown for 48 h in the presence of doxycycline (1.0 µg/mL), followed by preparation of native whole‐cell extract (WCE) and immunoprecipitation with FLAG Ab or IgG. Immunoprecipitates were electrophoresed for 30 min using 10% SDS‐polyacrylamide gels; upon entry of protein samples into separating gel, electrophoresis was terminated; gel slices were excised and processed for in‐gel trypsin digestion and sample preparation for LC‐MS/MS. (B) FLAG‐DDX5 interacting proteins identified by LC‐MS/MS analysis. (C) CORUM [31] prediction of significant complexes from (B). (D) Immunoblots of WCE from Dox‐inducible FLAG‐DDX5 expressing cells, grown without (−) or with (+) doxycycline (1.0 µg/mL) for 48 h. WCE immunoprecipitated with IgG or FLAG Ab, and immunoblotted with indicated antibodies. A representative experiment is shown from three independent experiments (*n* = 3) * indicates a contaminant detected with IgG. (E) HepaRG WCE native lysates immunoprecipitated with DDX5 Ab or IgG, followed by immunoblots with indicated antibodies (*n* = 2).

Our previous studies demonstrated that DDX5 acts as a host‐restriction factor for HBV biosynthesis [11]. To gain further insight into this mechanism of DDX5 action, we performed an LC‐MS/MS‐based proteomic study, using a Dox‐inducible cell line that expresses FLAG‐DDX5 (Figure 1A). Native whole‐cell extracts (WCE) were immunoprecipitated with FLAG antibody (Ab) or IgG, electrophoresed for 30 min in a 10% SDS polyacrylamide gel (Figure 1A), and following in‐gel digestion [30], peptides were analyzed by quantitative LC‐MS/MS. This LC‐MS/MS‐based proteomic analysis allows for high‐resolution separation of proteolytic peptides, enhancing chromatographic resolution and identification of greater number of peptides, including those of low abundance [30]. IFI16 and auxiliary PRC2 subunits RBBP4/7 were among the most significant DDX5 interacting proteins identified (Figure 1B).

To identify predicted complexes of DDX5 (Figure 1C), we utilized the Comprehensive Resource of Mammalian Protein Complexes (CORUM) database, comprised of more than 4000 mammalian complexes [31]. The top predicted complexes of DDX5 included well‐studied RNA processing complexes of DDX5 [32]; another DDX5 predicted complex involved the transcription silencing PRC2. Notably, our earlier studies [11] had identified the interaction between DDX5 and SUZ12, the core subunit of PRC2.

To validate the LC‐MS/MS results, lysates from the Dox‐inducible FLAG‐DDX5 expressing cell line, grown with or without Dox, were immunoprecipitated using FLAG Ab. FLAG‐DDX5 co‐immunoprecipitated with IFI16, RBBP4, and SUZ12, only in Dox‐treated cells (Figure 1D). Similarly, endogenous DDX5 also co‐immunoprecipitated with IFI16, RBBP4, and SUZ12 (Figure 1E), further supporting our previous finding that DDX5 interacts with SUZ12 [11].

**Figure 2:**
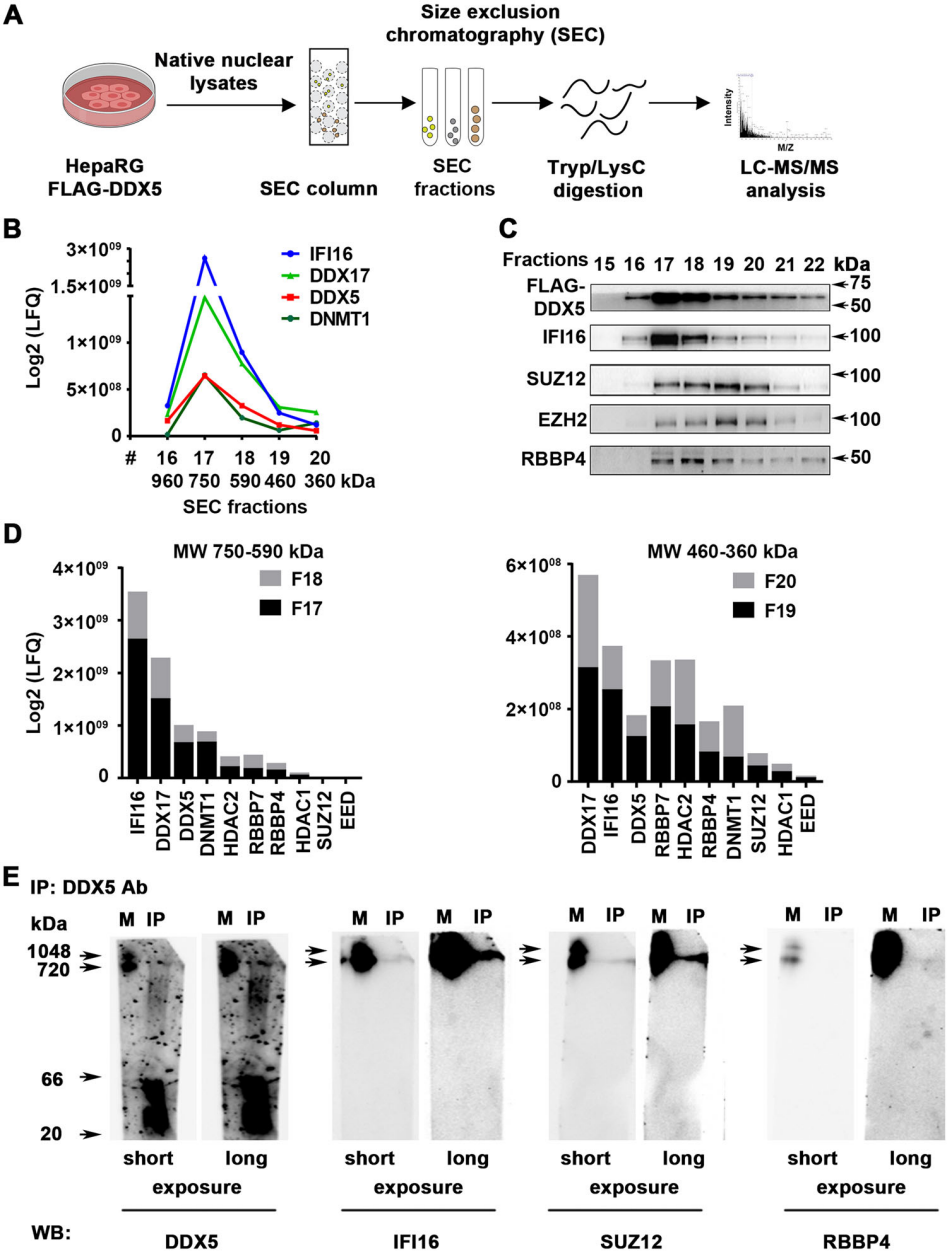
IFI16 co‐elutes with DDX5 in high MW protein complexes containing PRC2. (A) Workflow of nuclear protein complex identification by size exclusion chromatography (SEC). Nuclear lysates prepared under non‐denaturing conditions from FLAG‐DDX5 expressing HepaRG cells, fractionated by Superdex 200 10/300 GL column and AKTA fast protein LC system. (B) MW (kDa) of complexes eluting in indicated SEC fractions. (C) Immunoblots of SEC fractions (#15–22) for indicated proteins. (D) Label‐free quantification (LFQ) of indicated proteins identified by LC/MS/MS in fractions #17–20 (*n* = 2). (E) DDX5 immunoprecipitates, from HepaRG native nuclear lysates, analyzed by native polyacrylamide gel electrophoresis. Immunoblots of DDX5, IFI16, SUZ12 and RBBP4 are shown. Non‐specific binding of antibodies to unstained MW marker, visualized by coomassie blue staining of PVDF membrane. A representative assay is shown from *n* = 3.

To further validate these observations, we co‐fractionated native nuclear lysates from Dox‐inducible FLAG‐DDX5 expressing cells by SEC using Superdex 200 Increase gel filtration column (Figure 2A,B). SEC fractions spanning mass range from 750 kDa to less than 360 kDa were analyzed by immunoblots (Figure 2C) and LC‐MS/MS using Orbitrap Fusion Lumos MS (Thermo Fisher Scientific) (Figure 2D). The apparent molecular weight of proteins eluting in each SEC fraction was determined by column calibration with standard globular proteins ranging in molecular weight from 669 to 23 kDa.

DDX5 co‐eluted with IFI16, SUZ12, EZH2 the methyltransferase subunit of PRC2, and RBBP4, the auxiliary PRC2 subunit, in SEC fractions #17–22 (Figure 2C). LC‐MS/MS analysis of the collected fractions identified the most abundant proteins including IFI16, DDX17, a highly related RNA helicase [33], and DDX5. Several proteins with known transcription silencing activity, such as DNMT1 and HDAC1/2 were also detected (Figure 2B,D).

To confirm formation of this multicomponent DDX5/IFI16/PRC2 complex we employed two approaches. First, we performed native polyacrylamide gel electrophoresis of DDX5 immunoprecipitants using native nuclear lysates prepared from HepaRG cells expressing FLAG‐DDX5. This analysis revealed that the immunoprecipitated DDX5 complex migrated with a molecular weight of approximately 750 kDa, comprised of IFI16, SUZ12, and RBBP4 (Figure 2E).

**Figure 3:**
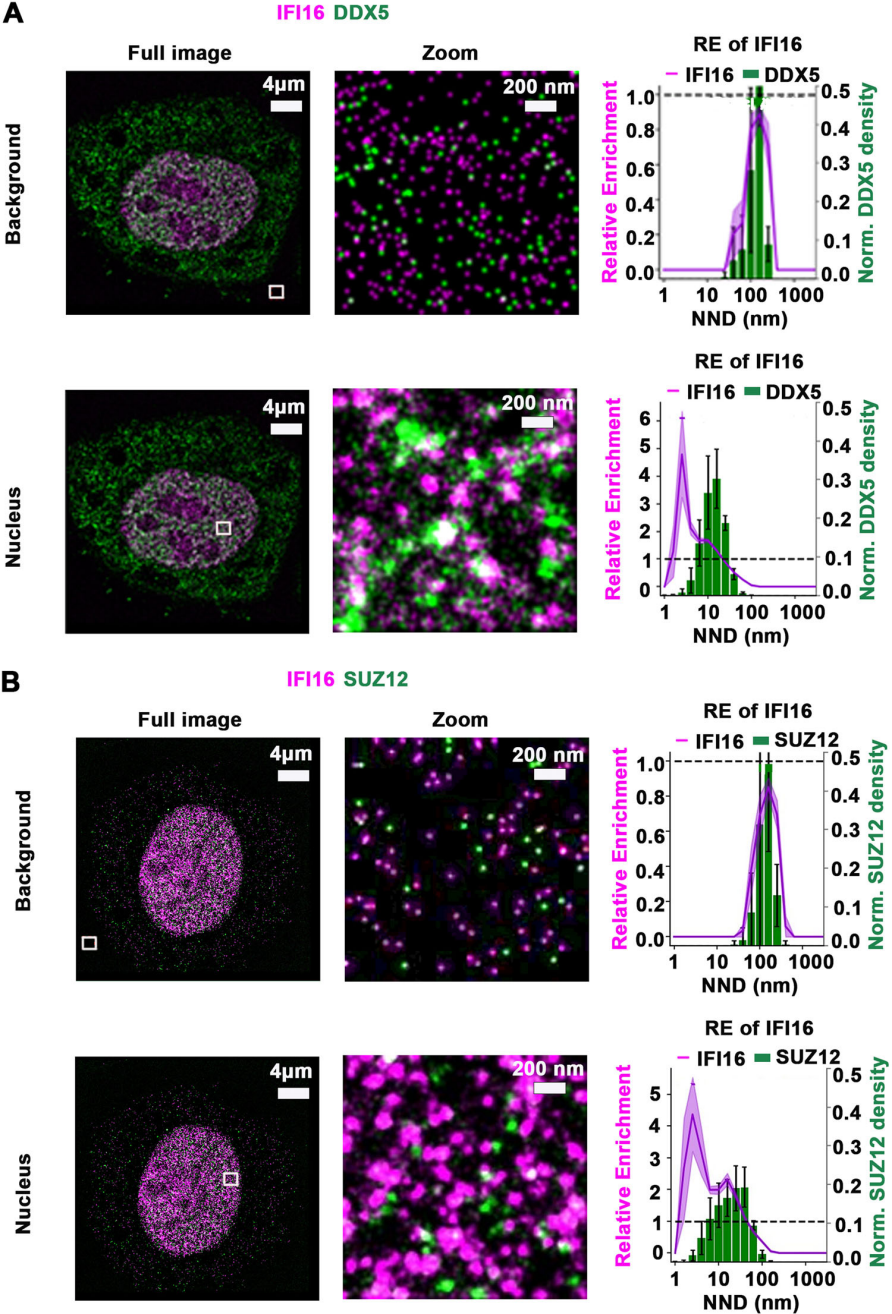
Visualizing relative enrichment (RE) of IFI16 across DDX5 and SUZ12 densities by SMLM. (A) Representative images by SMLM of IFI16 and DDX5, and (B) IFI16 and SUZ12 in HepaRG cells. Zoom sub‐regions, outlined by white lines, are from the background and nuclear areas. (Right panels) DDX5 regions binned by nearest neighbor distance (NDD in nm), with mean IFI16 RE value for each bin as a line plot. The graph shows RE score on the left‐hand *y*‐axis and the relative bin distribution on the right‐hand *y*‐axis. Purple shade and black bars indicate the standard error of the mean (SEM) from *n* = 8 images per cell, shown in Supporting Information S1: Figures S2–S5.

Second, we employed SMLM to determine the spatial localization of DDX5/IFI16/PRC2 in the cell nucleus (Figure 3). SMLM quantifications are coordinate‐based and colocalization cannot be evaluated by pixel‐intensity‐based approaches such as the Pearson coefficient. We therefore determined colocalization by calculating RE with Voronoi tessellation [31].

Specifically, RE of background fluorescence of 1.0 for IFI16 across DDX5 densities, binned by nearest neighbor distance (NND in nm), indicated uncorrelated distribution of the two molecules. In contrast, a mean RE > 1.0 of IFI16 in homogeneously stained regions in the nucleus, across the density distribution of DDX5 demonstrated the correlated distribution of IFI16 relative to DDX5 (Figure 3A and Supporting Information S1: Figures S1–S4). A similar analysis with dual‐color SMLM showed the correlated distribution of IFI16 and the core PRC2 subunit SUZ12 (Figure 3B and Supporting Information S1: Figures S5 and S6). Our data reveal RE at a length scale of 20 nm between the two protein species [34], supporting the co‐localization of IFI16 with DDX5 and PRC2 at nanoscale resolution.

### 3.1 Assembly of the DDX5 Complex With IFI16 Requires Interaction With RNA

**Figure 4:**
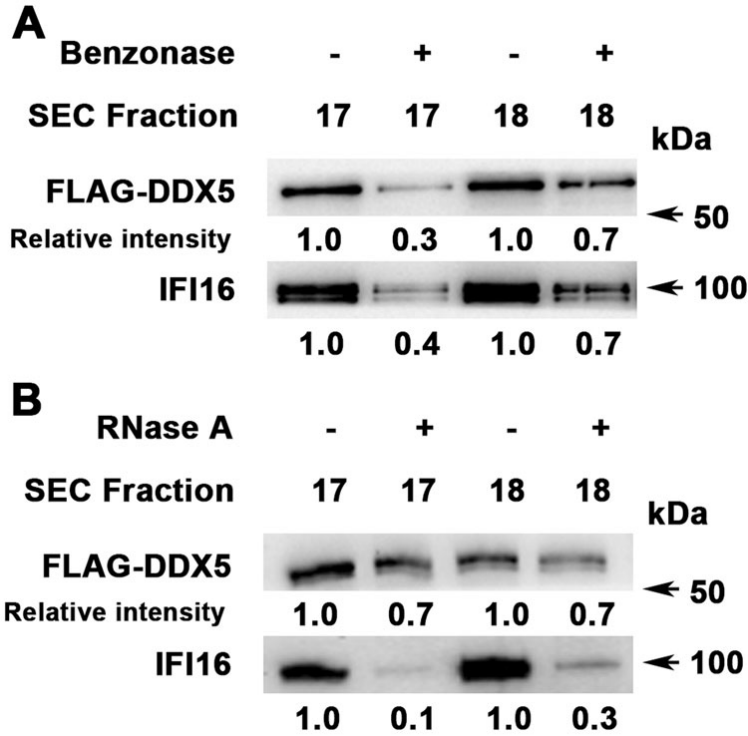
RNA is involved in the assembly of the IFI16/DDX5/PRC2 complex. Nuclear lysates prepared under non‐denaturing conditions from WT DDX5‐FLAG expressing cells treated with (+) or without (−): (A) benzonase, 125 U/mg lysate for 45 min on ice (*n* = 2) and (B) RNase A, 10 µg RNase A/mg of lysate at room temperature for 30 min, before SEC fractionation. Immunoblots of SEC fractions #17–18 with indicated antibodies. Relative intensity of DDX5 and IFI16 quantified by ImageJ software (*n* = 2).

Since DDX5 is an RNA‐binding protein, to determine whether the complex formation between DDX5 and IFI16 depended on RNA, we treated native nuclear lysates expressing WT FLAG‐DDX5 with the recombinant nuclease benzonase (Figure 4A) or RNase A (Figure 4B **).** This was followed by SEC analysis and immunoblotting. IFI16 exhibited significantly reduced association with WT FLAG‐DDX5 after treatment with benzonase or RNase A (Figure 4A,B **)**. Since DDX5 binds and remodels RNA, we interpret these results to indicate the involvement of an RNA molecule in the formation of the DDX5/IFI16/PRC2 complex. The identity of the RNA molecule is presently unknown.

### 3.2 The DDX5/IFI16/PRC2 Complex Restricts HBV by Directly Binding to cccDNA

**Figure 5:**
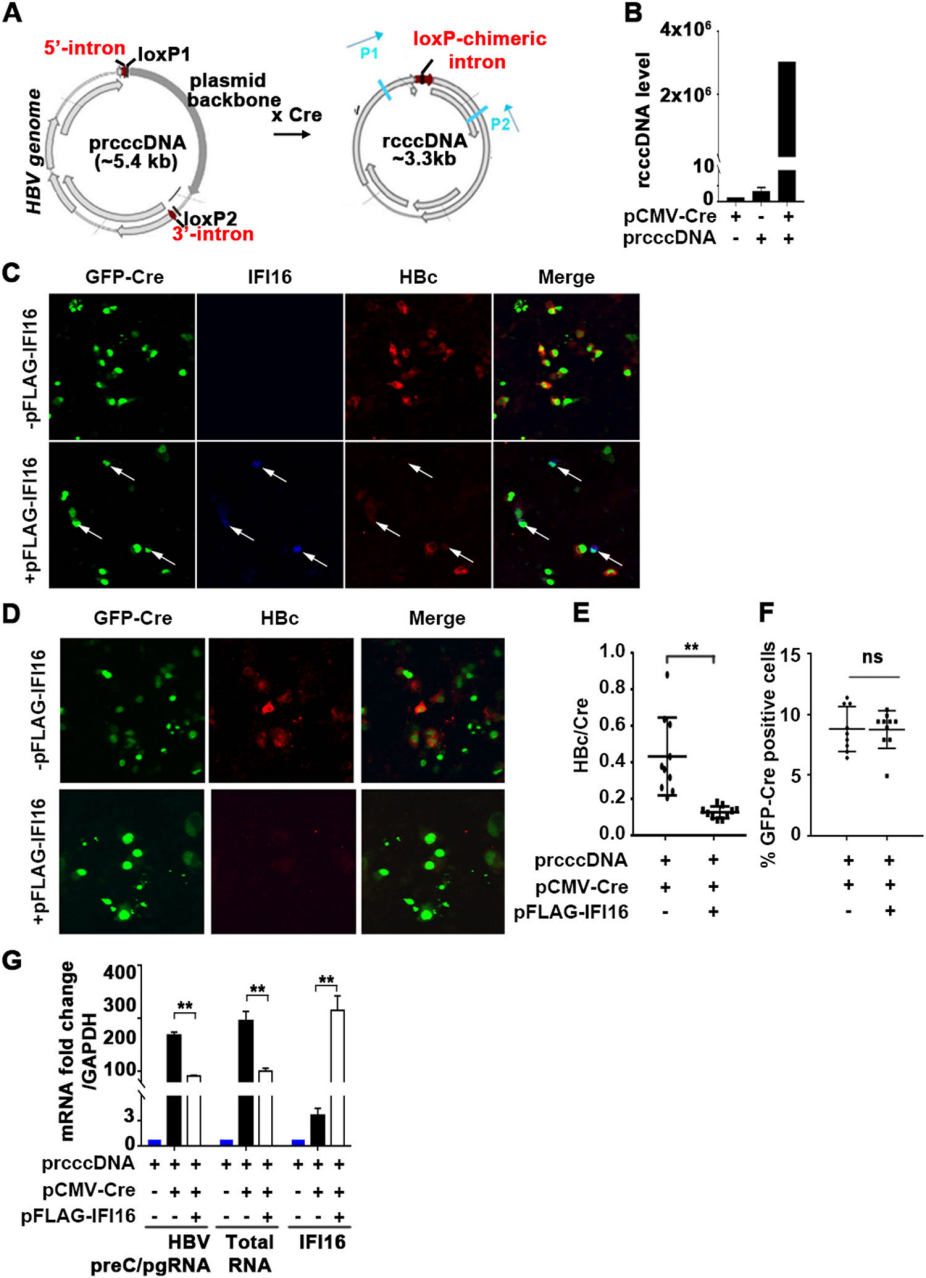
IFI16 represses viral gene transcription from a recombinant (r)cccDNA template of HBV. (A) Diagram, modified from Qi et al. [22], illustrates the generation of rcccDNA via Cre/loxP recombination. (B) PCR quantification of rcccDNA with P1/P2 primers using Hirts extracts of Huh7 cells transfected with indicated plasmids for 72 h. Data represent the average of two independent experiments. (C) Immunofluorescence microscopy of Huh7 cells co‐transfected for 72 h with prcccDNA and pCMVGFP‐Cre, with (+) or without (−) pCMV‐FLAG‐IFI16. Arrows indicate cells expressing IFI16 (blue), co‐staining with GFP‐Cre expressing cells, and HBc (red) expressing cells. (D–F) Immunofluorescence microscopy of Huh7 cells co‐transfected for 72 h with prcccDNA and pCMVGFP‐Cre, with (+) or without (−) pCMV‐FLAG‐IFI16. (E) Quantification of HBc/Cre and (F) GFP‐Cre‐positive cells, with (+) or without (−) IFI16 expression. Data are expressed as mean ± standard error of the mean (SEM) from *n* = 3. **p < 0.01 and ns = not significant. (G) qRT‐PCR quantification of viral RNAs (preC/pgRNA and total HBV RNAs) and IFI16 mRNA using RNA isolated from Huh7 cells, transfected with indicated plasmids for 72 h. Data are expressed as mean ± SEM from three independent experiments. **p < 0.01.

To determine the functional significance of the DDX5/IFI16/PRC2 complex on viral transcription, initially, we employed the recombinant rcccDNA system in Huh7 cells [22]. The precursor plasmid (prcccDNA) containing a loxP‐chimeric intron engineered into the HBV genome was co‐transfected with pCMV‐Cre recombinase in Huh7 cells, generating rcccDNA (Figure 5A) which forms chromatin‐like structure, similar to authentic HBV cccDNA [22]. Quantification of the rcccDNA was carried out by PCR, using the indicated P1 and P2 primers (Figure 5B).

Next, we investigated the effect of exogenous IFI16 expression on rcccDNA‐driven transcription. Huh7 cells were co‐transfected with prcccDNA and a plasmid encoding GFP‐Cre fusion protein, in the presence or absence of FLAG‐IFI16 expressing plasmid. GFP‐positive cells expressing Cre‐recombinase exhibited positive immunofluorescence (red) for the HBV core antigen HBc, in the absence of transfection with pFLAG‐IFI16. By contrast, only Cre‐GFP‐positive and transfected FLAG‐IFI16‐positive cells (blue) lacked immunostaining for HBc (Figure 5C). Next, we quantified the ratio of HBc/Cre‐positive cells with and without expression of IFI16. Exogenous IFI16 expression significantly reduced the ratio of HBc/Cre‐positive cells, without affecting transfection efficiency (Figure 5D–F). Likewise, exogenous IFI16 expression significantly reduced transcription of all HBV transcripts expressed from rcccDNA (Figure 5G **).** These results demonstrate that IFI16 represses transcription from rcccDNA, in agreement with earlier reports [21].

### 3.3 The DDX5/PRC2 Complex Mediates the Antiviral Effect of IFI16

**Figure 6:**
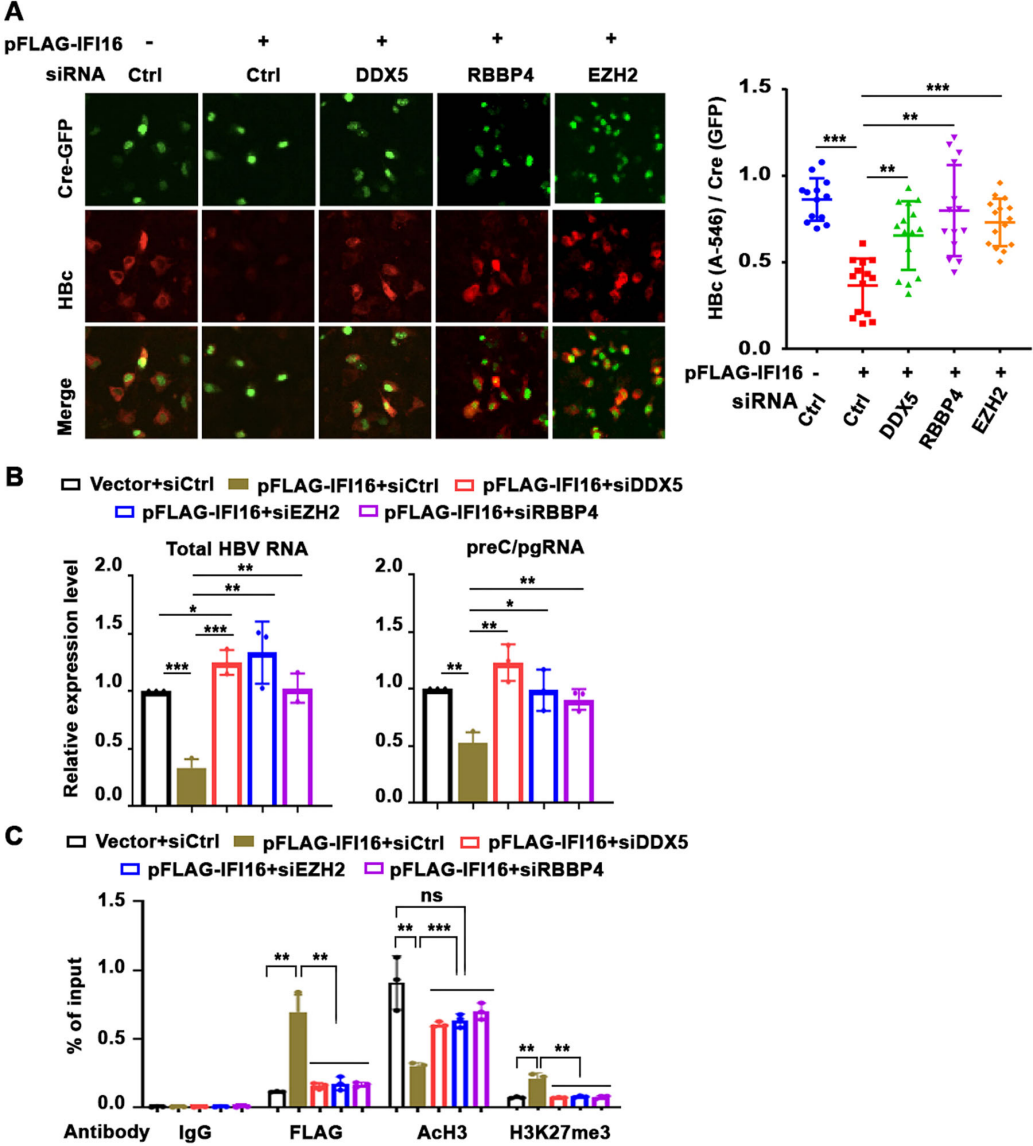
IFI16‐mediated repression of rcccDNA transcription is DDX5 and PRC2 dependent. (A) Immunofluorescence microscopy of Huh7 cells transfected for 72 h with prcccDNA and GFP‐Cre plasmids, without (−) and with (+) IFI16 encoding vector, and co‐transfected with 50 pM of indicated siRNAs. Quantification of HBc/Cre ratio from 1000 cells. Data are expressed as mean ± standard error of the mean (SEM) from *n* = 3. **p < 0.01, ***p < 0.001. (B) qRT‐PCR quantification of viral RNAs (total HBV RNA and preC/pgRNA) using RNA isolated from HepG2‐NTCP cells, transfected with indicated plasmids for 72 h. Data are expressed as mean ± SEM from three independent experiments. *p < 0.05, **p < 0.01, ***p < 0.001. (C) ChIP assays using IgG or indicated ChIP‐validated antibodies (FLAG, AcH3, and H3K27me3) and nuclear lysates from HepG2‐NTCP cells transfected for 72 h as in (A). Quantification of rcccDNA by PCR using P1 and P2 primers. Data are expressed as mean ± SEM from *n* = 3. **p < 0.01 ***p < 0.001, ns = not significant.

To investigate the functionality of the DDX5/IFI16/PRC2 complex in inhibiting rcccDNA‐driven transcription, we examined the effect of siRNA‐mediated knockdown of DDX5, RBBP4, and EZH2, in the presence of ectopic IFI16 expression. Employing fluorescence microscopy, we quantified the ratio of HBc/Cre‐GFP expressing cells (Figure 6A). Ectopic IFI16 expression significantly reduced the ratio of HBc/Cre‐GFP‐positive cells. In contrast, siRNA‐mediated knockdown of DDX5, RBBP4, or EZH2 in the presence of ectopic IFI16 expression restored the ratio of HBc/Cre expressing cells to nearly the level observed without IFI16. The effective siRNA‐mediated knockdown of DDX5, RBBP4 and EZH2, as well as the expression of IFI16 were confirmed by immunoblots (Supporting Information S1: Figure S7). To confirm these results, we quantified by RT‐qPCR the level of viral RNA transcripts following ectopic IFI16 expression, in the context of siRNA‐mediated knockdown of DDX5 and PRC2 subunits EZH2 and RBPP4 (Figure 6B). Indeed, FLAG‐IFI16 expression suppressed both total HBV RNA and preC/pgRNA levels, while siRNA knockdown of DDX5 and PRC2 subunits restored their expression (Figure 6B).

To directly demonstrate the functional role of the DDX5/IFI16/PRC2 complex in suppressing rcccDNA transcription, we performed ChIP assays, quantifying the levels of the activating H3 acetylation (AcH3) compared to the repressive H3K27me3 modification. Ectopic FLAG‐IFI16 significantly reduced AcH3 while increasing H3K27me3 levels. Importantly, siRNA‐mediated knockdown of DDX5, EZH2, or RBBP4 reversed these histone modifications of rcccDNA chromatin and reduced the binding of IFI16 to rcccDNA (Figure 6C and Supporting Information S1: Figure S8A). These results provide direct evidence for the functional involvement of the DDX5/IFI16/PRC2 complex in mediating the epigenetic modifications of the rcccDNA chromatin.

### 3.4 The DDX5/IFI16/PRC2 Complex Mediates the Antiviral Effect of IFN‐α on HBV Biosynthesis

**Figure 7:**
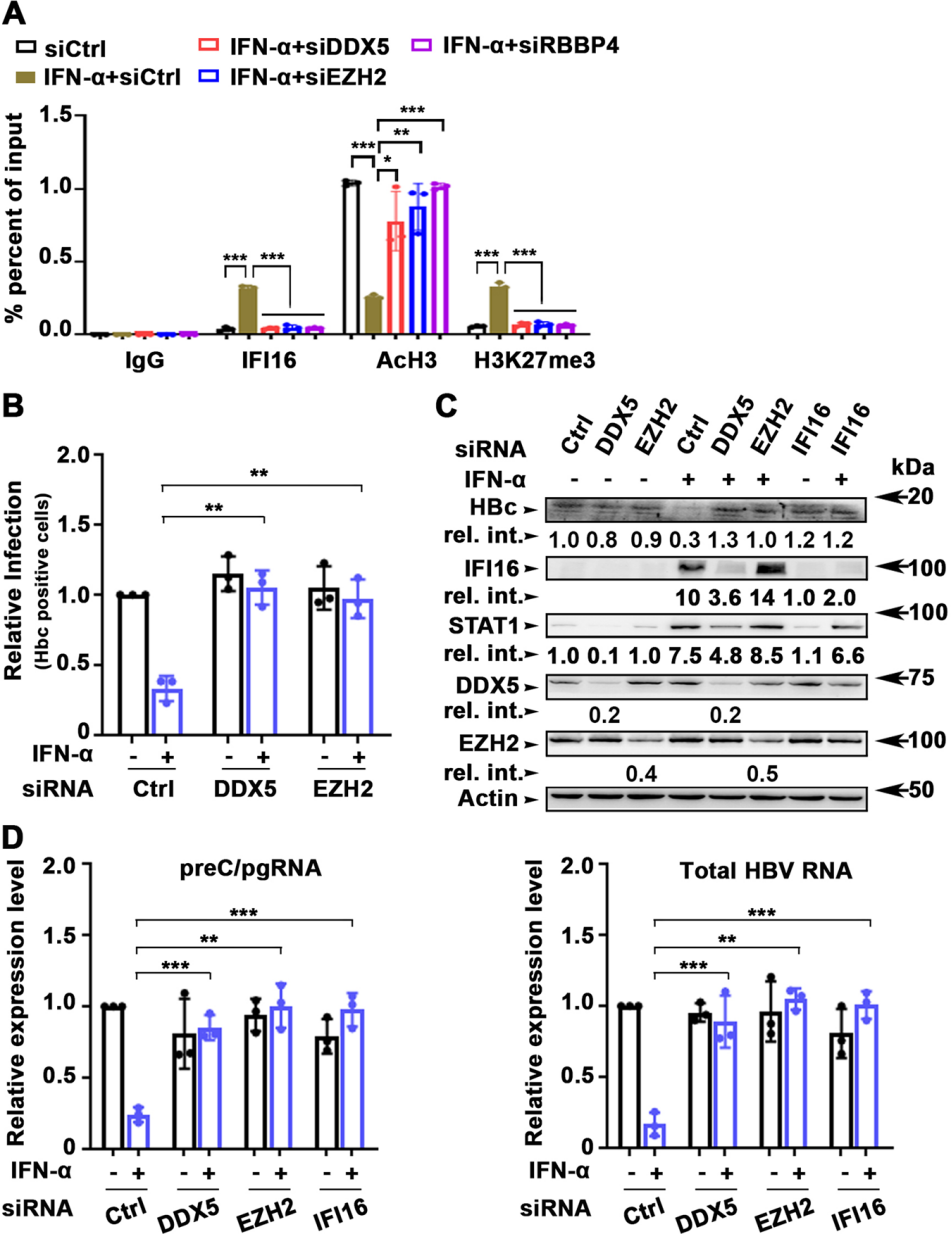
IFN‐α‐mediated inhibition of HBV infection is abrogated by DDX5 or PRC2 knockdown. (A) ChIP assays using IgG or indicated ChIP‐validated antibodies (IFI16, AcH3, and H3K27me3) and nuclear lysates from HepG2‐NTCP cells transfected for 72 h with prcccDNA and GFP‐Cre plasmids, without (−) and with (+) IFN‐α treatment, and co‐transfected with 50 pM of indicated siRNAs. Quantification of rcccDNA by PCR using P1 and P2 primers. Data are expressed as mean ± standard error of the mean (SEM) from *n* = 3. *p < 0.05 **p < 0.01 ***p < 0.001. (B) HepG2‐NTCP cells were transfected with 50 pM siRNA (siCtrl, siDDX5, siSUZ12, siEZH2, and siRBBBP4) on Days 0 and 4 p.i. HepG2‐NTCP cells infected with HBV (500 vge/cell), followed by daily addition (+) or not (−) of IFN‐α, were harvested on Day 8 p.i. Quantification of HBc‐positive cells by flow cytometry using anti‐HBc. Data are expressed as standard deviation (SD) from *n* = 3. **p < 0.01, ***p < 0.001. (C) Immunoblots of HBc, endogenous IFI16, STAT1, DDX5, and EZH2 using lysates from HBV infected cells on Day 8 p.i., treated as described in (B). A representative assay is shown from *n* = 3. (D) qRT‐PCR of preC/pgRNA and total HBV RNA isolated from HBV‐infected HepG2‐NTCP cells, treated as in (A). Data are expressed as mean ± SEM from *n* = 3. **p < 0.01, ***p < 0.001.

Recent studies indicate that IFI16 epigenetically represses transcription from the HBV cccDNA [21] by an unknown mechanism. Given that IFI16 is an interferon‐stimulated gene (ISG), induced by type I/II interferons [23], we investigated whether the DDX5/IFI16/PRC2 complex mediates the antiviral IFN‐α effect. To determine whether endogenous IFI16, induced by IFN‐α treatment, binds to cccDNA in a DDX5‐ and PRC2‐dependent manner, we conducted ChIP assays using HepG2‐NTCP cells transfected with prcccDNA/Cre plasmids and treated with or without IFN‐α for 72 h (Figure 7A). Our results show that endogenous IFI16 strongly binds to rcccDNA, in agreement with earlier reports [21]. Notably, siRNA‐mediated knockdown of DDX5, EZH2, or RBBP4 significantly diminished IFI16 binding to rcccDNA in IFN‐α‐treated cells. Additionally, the knockdown of DDX5 and PRC2 subunits reduced the H3K27me3 silencing modification linked to IFN‐α treatment, while increasing AcH3 levels associated with the rcccDNA (Figure 7A and Supporting Information S1: Figure S8A).

To further validate these findings, HepG2‐NTCP cells were infected with HBV and treated with or without IFN‐α for 8 days postinfection. Flow cytometry analysis revealed a reduction in the number of HBc‐positive cells following IFN‐α treatment (Figure 7B). Notably, siRNA‐mediated knockdown of DDX5 or EZH2 reversed the antiviral effects of IFN‐α without affecting cccDNA levels (Supporting Information S1: Figure S8B).

To assess the induction of endogenous IFI16 in HBV‐infected HepG2‐NTCP cells treated with IFN‐α, we analyzed lysates from the infected cells using immunoblotting (Figure 7C). The results indicated a decrease in HBc protein levels in cells treated with IFN‐α, while levels of IFI16 and STAT1 increased (Figure 7C). This treatment was also associated with downregulation of all viral RNAs, including preC/pgRNA and total HBV RNA (Figure 7D). Importantly, siRNA‐mediated knockdown of DDX5 or EZH2 abrogated the antiviral effect of IFN‐α, leading to increased number of HBc‐positive cells (Figure 7B), elevated levels of HBc protein (Figure 7C), and restoration of viral RNA expression in the presence of IFN‐α (Figure 7D). Similarly, the siRNA‐mediated knockdown of IFI16 abrogated the antiviral effect of IFN‐α on HBc protein levels (Figure 7C) and the expression of all viral RNAs (Figure 7D). Furthermore, in IFN‐α‐treated cells knockdown of DDX5 reduced expression of both IFI16 and STAT1 protein levels (Figure 7C), consistent with our earlier study demonstrating that DDX5 is a positive regulator of STAT1 translation [15]. Notably, STAT1 is known to induce IFI16 transcription in response to interferon [23, 24].

**Figure 8:**
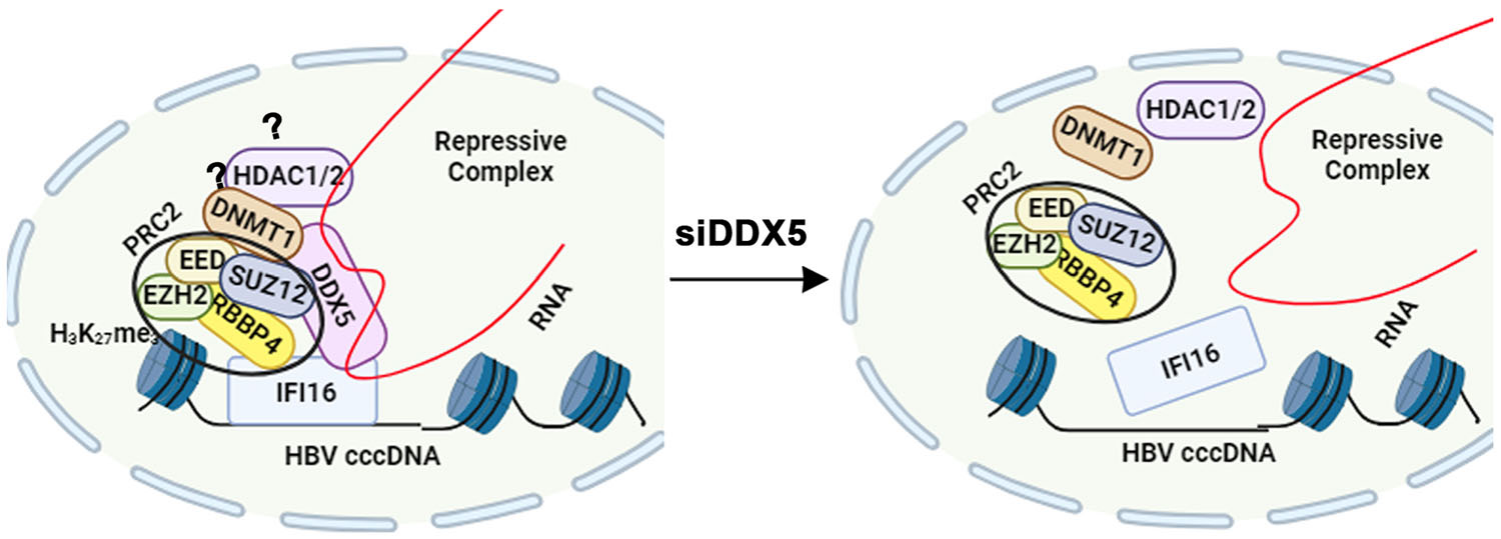
Diagram illustrates the IFI16‐directed binding of the DDX5/PRC2/RNA complex to cccDNA chromatin. Downregulation of DDX5, or loss of the associated RNA disassembles the complex, relieving IFI16 binding and the epigenetic repression of cccDNA transcription. The significance of DNMT1 or HDAC1/2 co‐eluting with the DDX5/IFI16/PRC2 complex, as suggested by the data of Figure 2D, remains to be determined.

Together, these findings support the involvement of the DDX5/IFI16/PRC2 complex in mediating the transcriptional silencing of the HBV mini‐chromosome in response to IFN‐α, and that downregulation of DDX5 exerts a proviral effect on HBV biosynthesis (Figure 8).

## 4 Discussion

In this study, we demonstrate the RNA helicase DDX5 restricts HBV transcription [11] by forming an epigenetic silencing multicomponent complex with IFI16 [35] and PRC2 [11]. Numerous host restriction factors have been identified for HBV [36], including the recently identified cohesin complex composed of SMC1 and SMC3 [37], HMGB1 [38], and the long noncoding RNA LINC01431 [39]. IFI16 has also been recently identified as a host nuclear DNA‐binding factor that restricts HBV cccDNA transcription epigenetically, by binding to the ISRE of the HBV cccDNA and increasing the transcription silencing H3K27me3 modifications [21]. However, the molecular mechanism by which IFI16 mediates this repressive H3K27me3 modification of cccDNA chromatin was not determined.

Using a proteomics approach, we identified IFI16 as the most prominent DDX5 interacting protein (Figure 1). Interrogating the LC‐MS/MS results with the CORUM database [31] predicted several epigenetic silencing complexes interacting with DDX5, notably PRC2, which mediates the transcription silencing H3K27me3 modification (Figure 1). This prediction agrees with our earlier observations that DDX5 interacts with SUZ12, the core subunit of PRC2 [11]. Importantly, IFI16 co‐immunoprecipitated with DDX5 and PRC2 subunits, strongly suggesting that these proteins form a multicomponent complex. LC‐MS/MS profiling of SEC fractions from native nuclear lysates demonstrated that DDX5 co‐eluted with IFI16, DDX17, DNMT1, HDAC1/2, RBBP4/5, and EZH2/SUZ12 of PRC2 (Figure 2). DDX5 heterodimerizes with DDX17, a related RNA helicase [33]. Interestingly, helicase‐active DDX17 binds the 5′ epsilon structure of HBV pgRNA inhibiting its encapsidation and acting as another host‐restriction factor against HBV [40]. It is tempting to speculate that DDX17 contributes to the antiviral IFN‐α effect by inhibiting pgRNA encapsidation, in association with the DDX5/IFI16/PRC2 complex. Recent studies have also elucidated an additional function of DDX5 and DDX17 helicases in restricting HBV biosynthesis, namely disruption of viral transcription termination leading to transcriptional readthrough [41]. This causes HBV RNA destabilization and decreased HBx protein expression [41]. Whether DNMT1 or HDAC1/2 (Figure 2D) are also components of the DDX5/IFI16‐based multicomponent complex remains to be determined. Similarly, it remains to be determined whether TRIM21 [42] and nucleolin [43] are components of this complex. The E3 ligase TRIM21 may facilitate the degradation of IFI16 [42], while nucleolin is known to bind with G‐quadruplexes in DNA or RNA [43]. Our unpublished observations suggest that this DDX5‐based epigenetic complex targets the silencing of cellular promoters that contain G‐quadruplexes. We hypothesize that this silencing may occur via IFI16, which preferentially binds to DNA with G‐quadruplex structures [44]. Alternatively, DDX5‐mediated epigenetic silencing could also involve nucleolin, given its capacity to bind G‐quadruplex structures [43]. Further research is needed to explore these hypotheses.

The DDX5/IFI16/PRC2 complex was detected by native polyacrylamide gel electrophoresis, migrating with a molecular weight of nearly 750 kDa, consistent with our SEC fractionation studies (Figure 2). Additionally, super‐resolution microscopy confirmed the co‐localization of DDX5, IFI16, and SUZ12 of PRC2 at nanoscale resolution (Figure 3). Our findings also indicate the involvement of a yet unknown RNA molecule in the assembly of the DDX5/IFI16/PRC2 complex. The evidence in support of this model includes the reduction in complex formation upon treatment of native nuclear lysates with benzonase or RNase A (Figure 4). Regarding known long‐noncoding RNAs (lncRNAs) that regulate cccDNA transcription, lncRNA HOTAIR in association with DDX5/PRC2 was shown to repress HBV cccDNA transcription [11]. Recent studies have identified LINC01431 as an epigenetic remodeler of the HBV mini‐chromosome by interacting and stabilizing PRMT1, which increases H4R3me2a and reduces cccDNA histone acetylation [39]. Conversely, lncRNA Dleu2 induced by HBx is a positive effector of HBV replication [45]. Thus, expression of specific lncRNAs exerts distinct effects on cccDNA transcription, namely repression [40] versus activation [42]. We propose that global epigenomic analyses are needed to identify lncRNAs that form the DDX5/IFI16/PRC2 epigenetic silencing complex, particularly under conditions of antiviral immunity, since this DDX5/IFI16/PRC2 complex mediates the antiviral IFN‐α effect (Figure 7).

IFI16 is an ISG [23], and DDX5 positively regulates STAT1 translation [15], a key transcription factor mediating all types of interferon signaling [46]. In IFN‐α treated HBV infected cells, siRNA‐mediated knockdown of DDX5 reduced protein levels of STAT1 and endogenous IFI16, and restored viral gene transcription (Figure 7), linking DDX5 function to immune signaling and viral gene expression. Specifically, induction of endogenous IFI16 by IFN‐α transcriptionally silences cccDNA, while siRNA‐mediated knockdown of DDX5 or PRC2 subunits abrogates the IFI16 effect (Figure 7). We propose that the DDX5/IFI16/PRC2 complex mediates the antiviral IFN‐α effect on cccDNA transcription (Figure 8).

Much remains to be understood about the regulation of this DDX5/IFI16/PRC2 silencing complex, and how HBx overcomes its restrictive function. One possibility is that HBx‐induced DLeu2 lncRNA [45] competes and interferes with DDX5/IFI16/PRC2 complex formation, by directly binding EZH2. Alternatively, posttranslational modifications induced by HBx [47, 48, 49] could regulate the assembly of this DDX5/IFI16/PRC2 complex. Our preliminary studies have identified phosphorylation of DDX5 in vitro and in vivo, as well as in vivo phosphorylation of IFI16 at Ser/Thr sites (data not shown). Additionally, in vivo methylations of IFI16 at both Lys and Arg residues have been identified, likely mediated by EZH2 or PRMT1 (data not shown). The functional significance of these modifications remains to be understood.

Similarly, the identification of the RNA involved in the assembly of the IFI16/DDX5 silencing complex, and its expression during HBV infection will provide better understanding of factors determining viral persistence. HBV persistence is associated with the continuing presence of transcriptionally active cccDNA in infected hepatocytes, resulting in continuous viral replication. HBV persistence is also associated with inhibition of innate immune signaling [50]. Chronic HBV patients have reduced expression of type I interferon genes, including IFI16 [21], and much higher cccDNA transcription [50]. Since the downregulation of DDX5 abrogates the antiviral INF‐α effect (Figure 7) by regulating STAT1 translation and IFI16 expression, as well as the formation of the RNA‐based DDX5/IFI16/PRC2 complex, we propose that DDX5 orchestrates HBV cccDNA transcription.

Our working model suggests that DDX5 recognizes and binds to specific RNA/RNA secondary structures [14], recruits IFI16, PRC2, and other epigenetic enzymes [51], partitioning/demixing these proteins in biomolecular condensates to influence cccDNA transcription [52]. This DDX5‐dependent complex and its function may also modify the nearby cellular chromatin, thereby influencing cellular gene expression and HBV disease pathogenesis. Recent studies have demonstrated that IFI16 is involved in liquid–liquid phase separation (LLPS) in the context of HSV1 infection and cytokine gene expression [53]. Further studies are needed to determine whether this DDX5‐based IFI16/PRC2 complex forms biomolecular condensates involved in LLPS, in the context of HBV infection and IFN‐α treatment of chronically HBV‐infected patients.

## Author Contributions

Zhili Li performed the experiments shown in Figures 2E, 3, 6B,C, and 7. Naimur Rahman performed experiments shown in Figures 1, 2A–D, 4, 5, and 6A. Fang Huang and Cheng Bi performed the super‐resolution studies (Figure 3). Uma K. Aryal and Rodrigo Mohallem directed and assisted with proteomic studies. Aryamav Pattnaik and Majid Kazemian assisted with flow cytometry (Figure 7). Ourania Andrisani directed the study and wrote the manuscript.

## Ethics Statement

Ethics approval obtained by Purdue University (Ref. ID #97‐010‐21).

## Consent

The authors have nothing to report.

## Conflicts of Interest

The authors declare no conflicts of interest.

## Supporting information

Supporting information.

## Data Availability

The data that support the findings of this study are openly available. Raw LC‐MS/MS data files can be accessed through MassIVE (https://massive.ucsd.edu/) with the ID: MSV000091599 https://massive.ucsd.edu/ProteoSAFe/dataset.jsp?task=d8f25ea194f14c679539dc186d9f7790.
