# Supplementary material for: RNA Helicase DDX5 in Association With IFI16 and the Polycomb Repressive Complex 2 Silences Transcription of the Hepatitis B Virus by Interferon
Source: J Med Virol. 2024 Dec 16;96(12):e70118. doi: 10.1002/jmv.70118 (PMC11648352; doi:10.1002/jmv.70118)
Supplement: Supplementary file 1 — Supporting information. [file JMV-96-e70118-s001.docx]

**RNA helicase DDX5 in association with IFI16 and the Polycomb Repressive Complex 2 silences transcription of the Hepatitis B virus by interferon.**

Zhili Li^1,2^, Naimur Rahman^1,2^**,** Cheng Bi^3^, Rodrigo Mohallem^4,5^ Aryamav Pattnaik^2,6^, Majid Kazemian^2, 6, 7^, Fang Huang^3^, Uma K. Aryal^4,5^ and Ourania Andrisani^1, 2*^

^1^Department of Basic Medical Sciences, ^2^Purdue Institute for Cancer Research,^3^Department of Biomedical Engineering, ^4^Purdue Proteomics Facility, Bindley Bioscience Center, ^5^Department of Comparative Pathobiology, ^6^Department of Biochemistry, ^7^Department of Computer Science,

*corresponding author: [andrisao@purdue.edu](mailto:andrisao@purdue.edu)

Department of Basic Medical Sciences,

Purdue University

201 S. University Street

West Lafayette, IN  47907-2064

Phone: 765-494-8131

**Supplementary Figures**

**
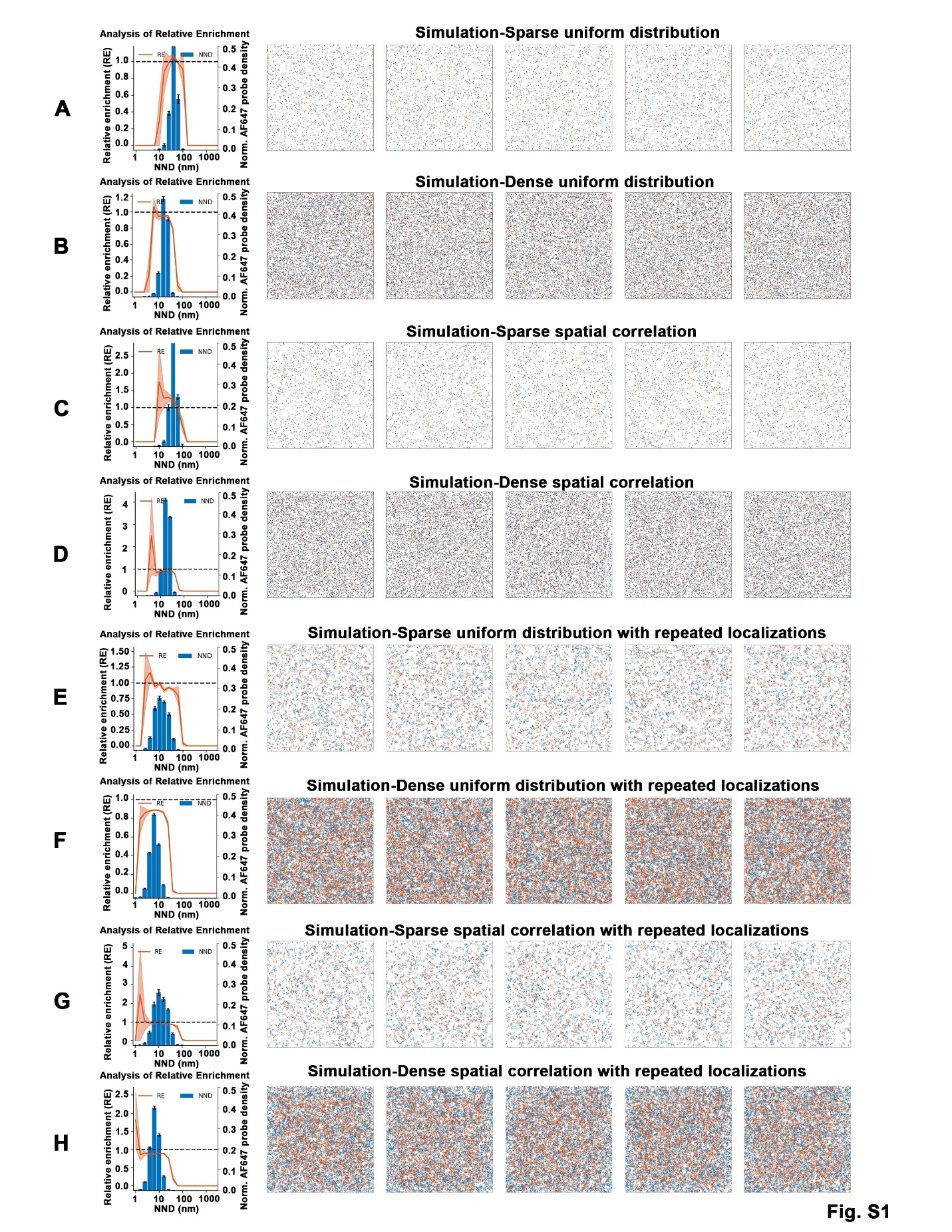
**

**Figure S1. Simulation of two molecular species testing relative enrichment method**

Simulation of two uniformly distributed molecular species with different molecular density using MATLAB. The curve shows the mean RE value of the primary species (orange), calculated from 5 different simulations, with the shaded area representing the standard error of the mean. The histogram shows the mean nearest neighbor distance of the reference species (blue). In both sparse and dense scenarios, uniformly distributed molecules yield RE values close to 1 (A, B). Meanwhile, simulations of spatially correlated molecules result in RE values larger than 1 (C, D). Further analysis, considering repeated localizations, confirms that the RE values remain consistent, regardless of the presence of repeated localizations (E, F, G, and H)

**
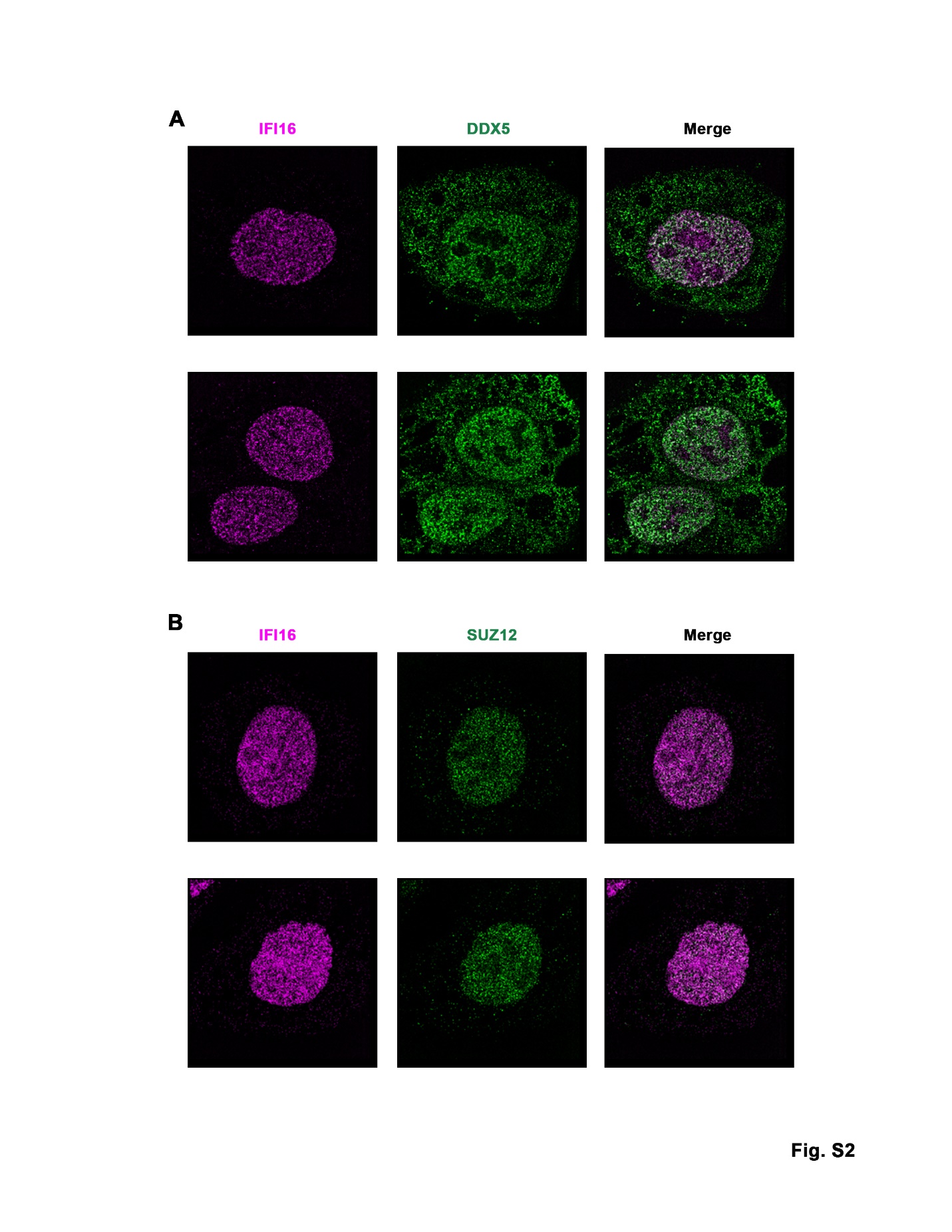
**

**Figure S2. (A)** Super-resolution images of IFI16 and DDX5, and **(B)** IFI16 and SUZ12, in HepaRG cells.

**
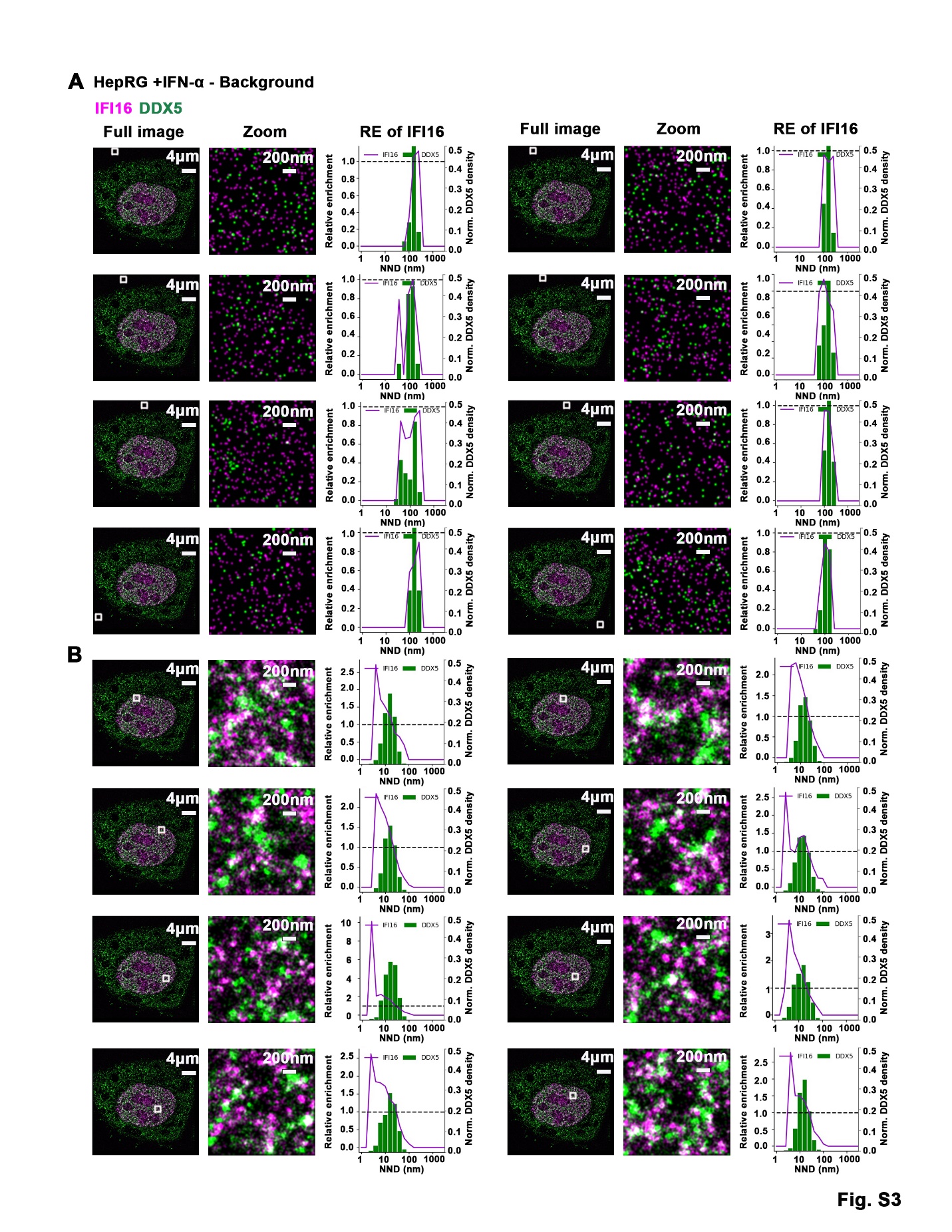
**

**Figure S3. Visualizing relative enrichment (RE) of IFI16 across DDX5 densities by SMLM.** Representative images by SMLM of IFI16 and DDX5. Zoom sub-regions are from indicated boxed background (A) and nuclear areas (B). (Right panels) DDX5 regions binned by nearest neighbor distance (NDD, nm), with IFI16 RE value for each bin as line plot. Graph shows RE score on left-hand y-axis and relative DDX5 density on right-hand y-axis per quantification.


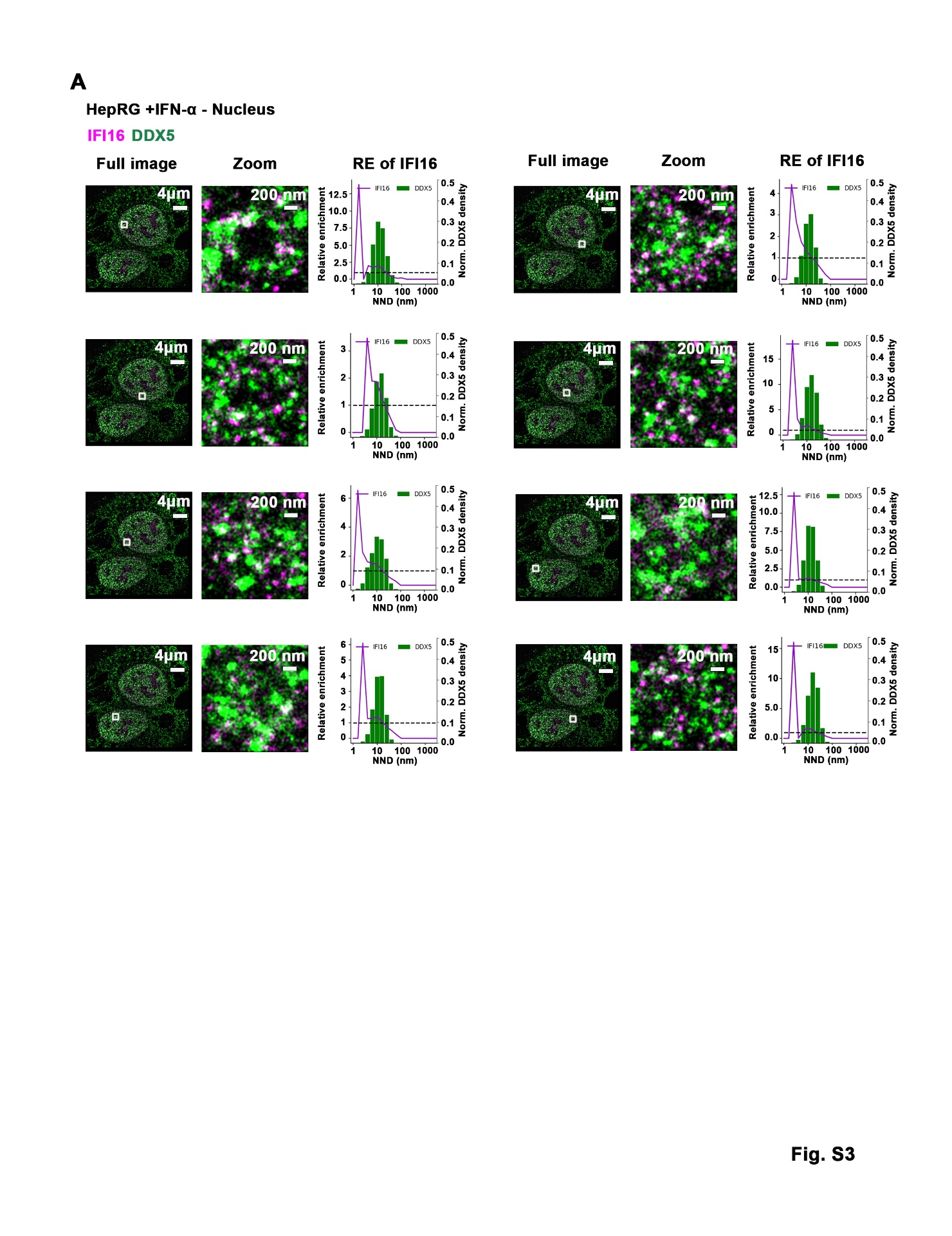


**Fig. S4**

**Figure S4. Visualizing relative enrichment (RE) of IFI16 across DDX5 densities by SMLM.** Representative images by SMLM of IFI16 and DDX5. Zoom sub-regions are from indicated boxed nuclear areas. (Right panels) DDX5 regions binned by nearest neighbor distance (NDD, nm), with IFI16 RE value for each bin as line plot. Graph shows RE score on left-hand y-axis and relative DDX5 density on right-hand y-axis per quantification.

**
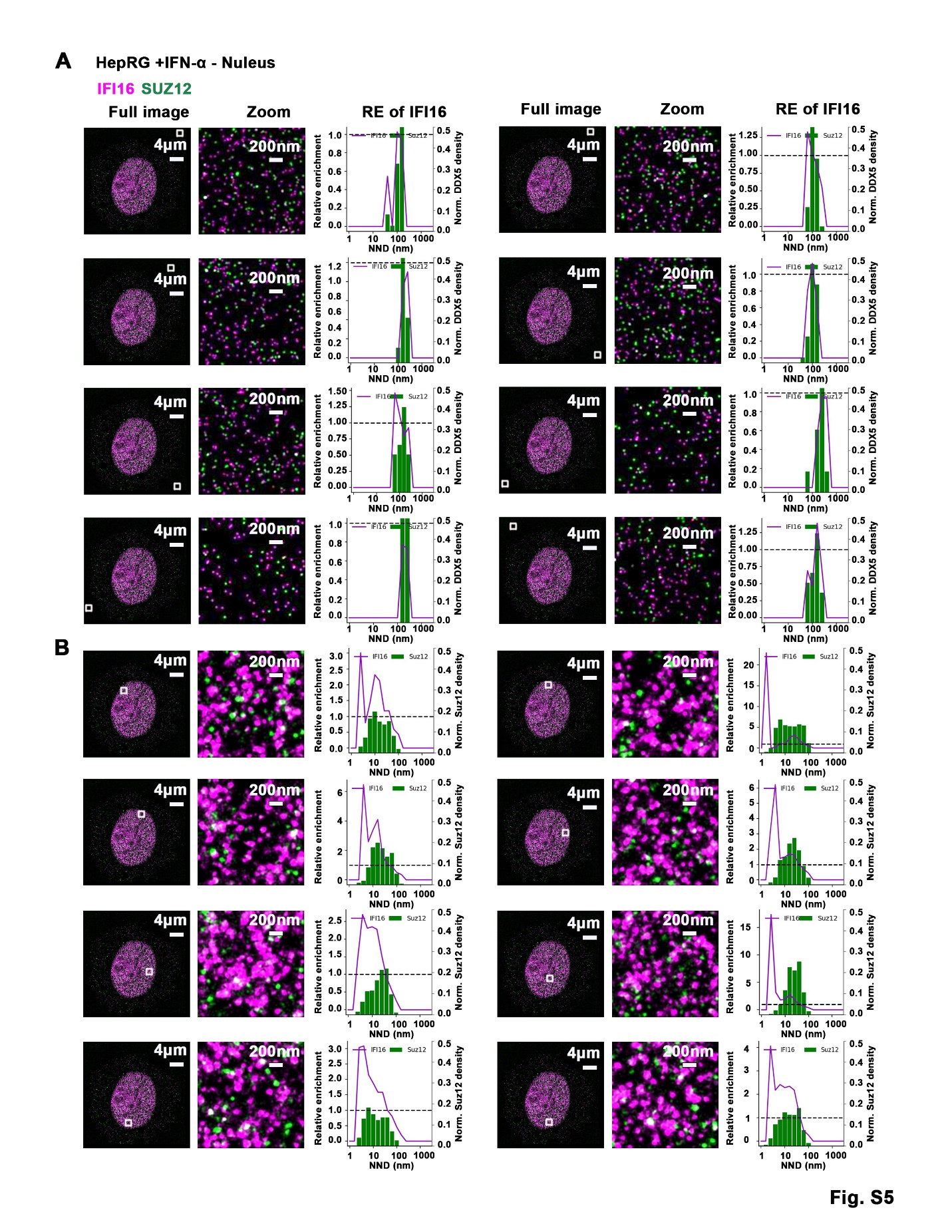
**

**
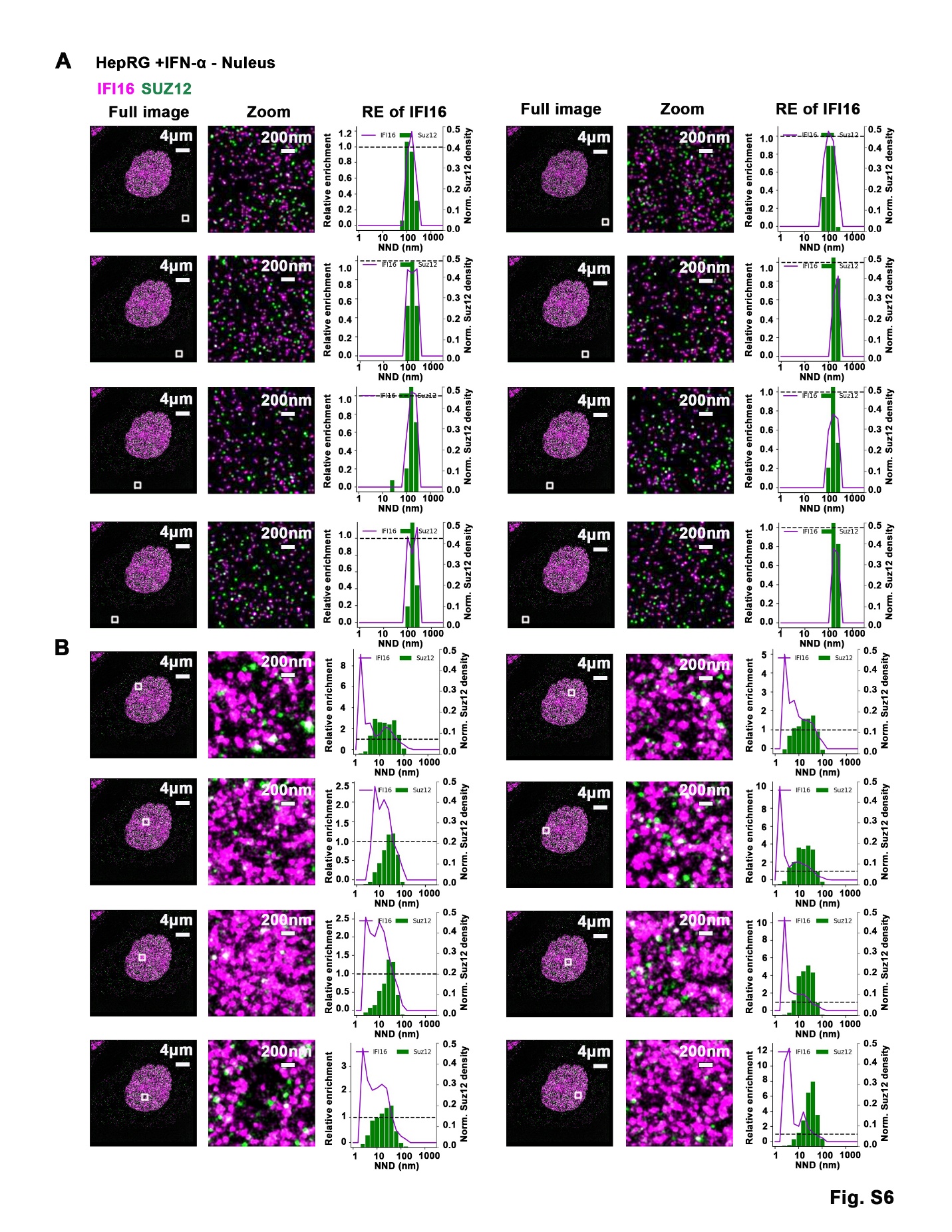
**

**Figures S5 and S6.** Visualizing relative enrichment (RE) of IFI16 across SUZ12 densities by SMLM**.** Representative images by SMLM of IFI16 and SUZ12. Zoom sub-regions are from indicated boxed background (A) and nuclear areas (B). (Right panels) SUZ12 regions binned by nearest neighbor distance (NDD, nm), with IFI16 RE value for each bin as line plot. Graphs show RE score on left-hand y-axis and relative SUZ12 density on right-hand y-axis per quantification. **Figures S5 and S6** analyzed two different cells.


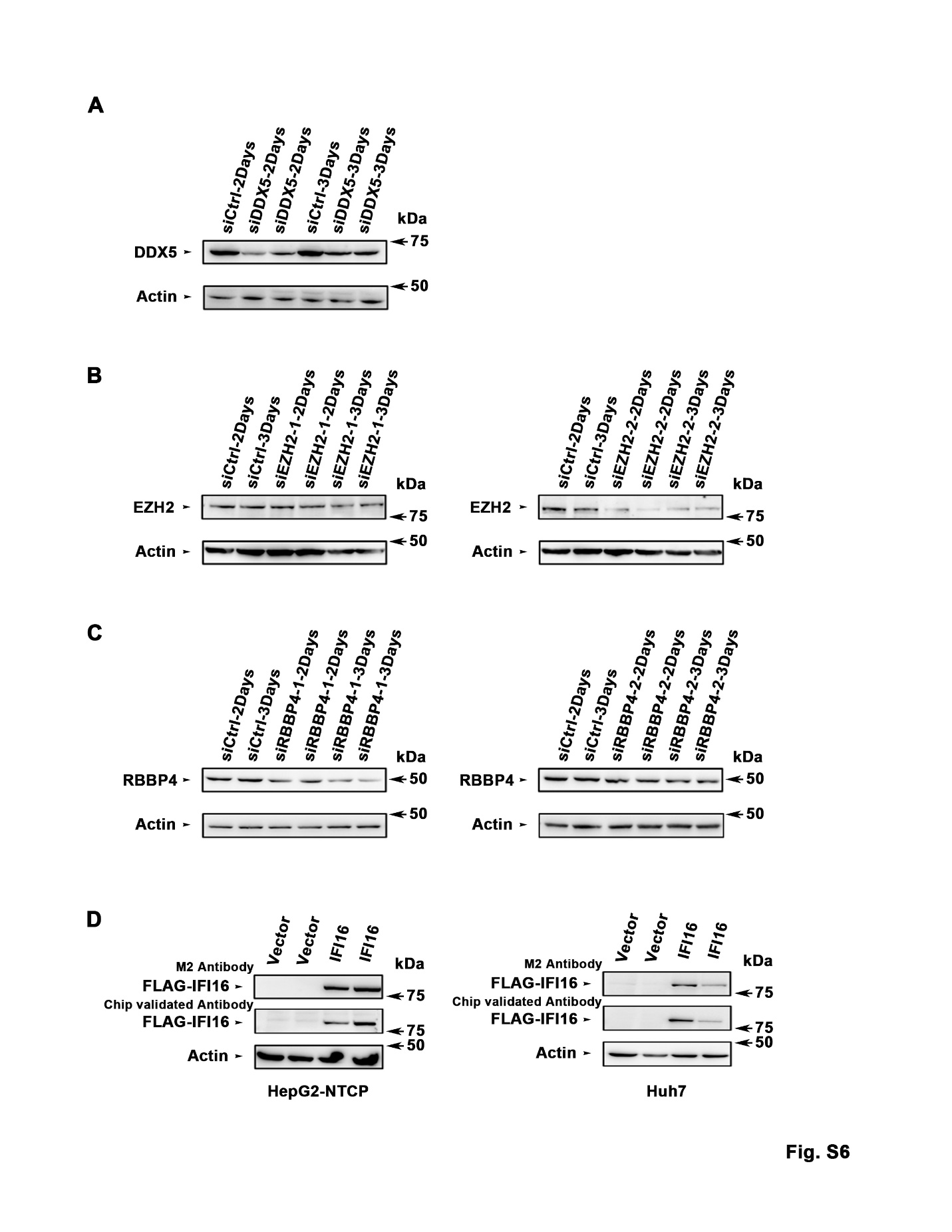


**Fig. S7**

**Figure S7.** (**A-C**) Immunoblots of indicated proteins, testing the knockdown efficacy of siRNAs transfected at 50pM in Huh7 cells for 2 and 3 days. B and C. show the efficacy of siRNA-1 and siRNA-2 for EZH2 (**B**), and RBPP4 (**C**). **(D)** pFLAG-IFI16 transfected in Huh7 in HepG2-NTCP and Huh7 cells. IFI16 expression detected by immunoblots using the FLAG M2 antibody (Sigma) or the ChIP-validated FLAG antibody (Cell Signaling).

**
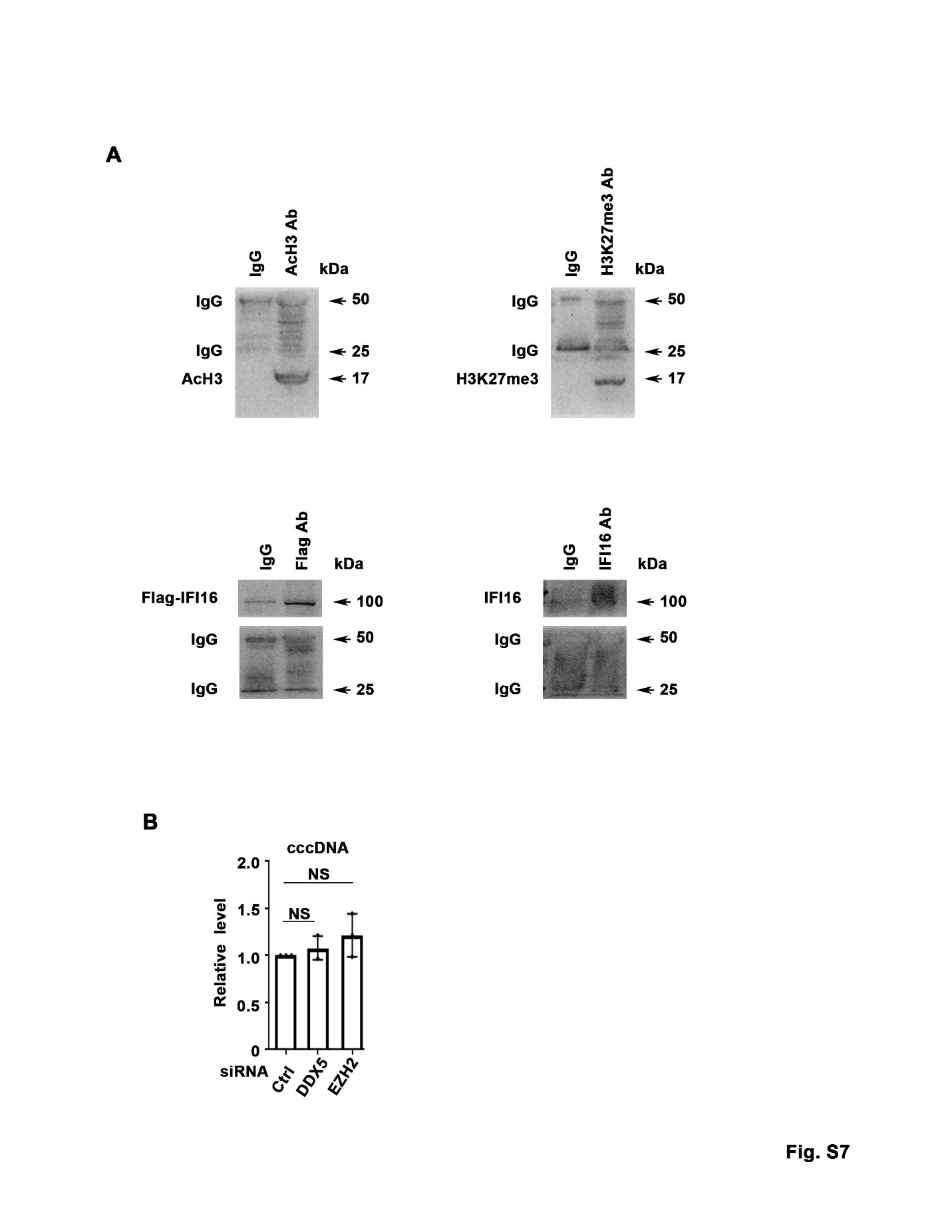
Fig. S8**

**Figure S8.** (**A**) Immunoblots of indicated proteins, testing the immunoprecipitation efficiency of antibodies for AcH3, H3K27me3, FLAG and IFI16 vs. IgG used in the ChIP assays of Figs.6C and 7A. **(B)** PCR cccDNA quantification, on day3 of HBV-infection. HepG2-NTCP cells were transfected with indicated siRNAs on day 0 prior to HBV infection. cccDNA purification and PCR quantification performed exactly as described (29). Data are expressed as Standard Deviation (SD) from n=3. NS=not significant.

**Supplementary Materials and Methods**

**Whole cell extracts preparation for LC-MS/MS:** Whole cell extracts were prepared from doxycycline inducible HepaRG-FLAG-DDX5 cell lines. Approximately 2x10^6^ cells were seeded in 100 mm dishes. The following day, cells were treated with doxycycline (1.0 µg/ml) for 48 h. Cells were washed 3X and collected by scraping in ice-cold phosphate-buffered saline (PBS), followed by centrifugation for 5 min at 500 x g. Cells pellets were lysed in 1x cell lysis buffer (Cell Signaling), sonicated on ice for 30 secs and clarified by centrifugation at 13,000 rpm for 15 min. at 4^0^C. Protein concentration was determined by bicinchoninic acid (BCA) assay (Pierce Chemical Co., Rockford, IL, USA).

**Immunoprecipitation (IP) for LC/MS/MS:** For immunoprecipitation of FLAG-DDX5, 1.0 mg of protein lysate was incubated with 30 μl of Anti-FLAG magnetic agarose beads (Thermo Fisher Scientific) overnight at 4 °C. Equal amount of protein lysate (1.0 mg) was incubated with IgG for 1h at 4 °C, followed by addition of protein A/G-agarose beads (Sigma) overnight at 4 °C. Subsequently, the beads were washed three times with Tris-buffered saline containing 0.05% Tween-20. Immunoprecipitated proteins were eluted by boiling for 5 min in 1× SDS Laemmli buffer and electrophoresed for 30 min on 10% SDS polyacrylamide gels.

**Native nuclear extract preparation:** Nuclear extracts were prepared from doxycycline inducible HepaRG-FLAG-DDX5 cell lines, grown with doxycycline (1.0 µg/ml) for 48 h. Cells were washed 3X and collected by scraping in ice-cold phosphate-buffered saline (PBS), followed by centrifugation for 5 min at 450 x g. Cell pellets resuspended in hypotonic lysis buffer (10 mM HEPES pH 7.9, 1.5 mM MgCl2, 10 mM KCl, 1 mM dithiothreitol), were incubated on ice for 15 min and collected by centrifugation at 420xg for 5 min ; pellets resuspended in hypotonic lysis buffer, homogenized on ice for ten strokes (type B pestle), and centrifuged at 3000 x g for 20 min. The pellet was resuspended in extraction buffer, containing 20 mM HEPES pH 7.9, 25% glycerol, 420 mM NaCl, 0.2 mM EDTA, 1 mM DTT and proteinase inhibitors, homogenized on ice with ten strokes (type B pestle), followed by incubation for 30 min at room temperature with gentle shaking. Chromatin pelleted by centrifugation at 20,000x g for 5 min at 4°C, and soluble nuclear protein lysate used immediately for size exclusion chromatography.

**Size Exclusion Chromatography (SEC):** Native nuclear lysates were separated on Superdex 200 10/300 GL column (GE Healthcare) using an ÄKTA fast protein liquid chromatography (FPLC) system (Amersham Biosciences). SEC column was equilibrated with buffer A (50 mm Tris-HCl, pH 7.5) overnight. Column calibration was performed using protein standards (MW GF1000, Sigma-Aldrich) ranging in molecular weight from 669 kDa to 23 kDa. A total of 400 μl nuclear lysate (1.0 mg protein) was loaded onto the column and eluted in 1.25 column volumes at a flow rate of 0.2 mL/min. Void volume was measured with blue dextran. SEC separation performed at 6 °C. Fractions analyzed by LC–MS/MS.

**Proteomics Sample Preparation of SEC fractions.** Fifty (50) µg protein from each SEC fraction was precipitated with 4 volumes of cold (-20°C) acetone and incubated overnight at -20°C. Samples centrifuged at 13,500 rpm for 15 minutes at 4°C, supernatant (acetone) discarded, and pellet dried in vacuum. Dried protein pellets were resuspended in 10 µl of 10 mM DTT in 8M urea and incubated at 37 °C for 1 h. Equal volume of alkylating mixture (195 µl of 97.5% acetonitrile, 1.0 µl of 0.5% triethyl phosphate and 4.0 µl of 2% iodoethanol) was added, samples were incubated at 37 °C for 1 h before drying in vacuum. Samples were digested with Lys-C/Trypsin in 25 mM ammonium bicarbonate in a 1:25 enzyme to protein ratio, using a Barocycler (60 cycles: 20k PSI for 50 seconds and 1 ATM 10 seconds, at 50 °C). Digested peptides were desalted using C_18_ Silica MicroSpin Columns (The Nest Group, Inc. USA).

**Liquid Chromatography-Tandem Mass Spectrometry (LC-MS/MS) analysis:** Digested peptides were analyzed on Orbitrap Fusion Lumos Mass Spectrometer (Thermo Fisher Scientific) equipped with a Dionex UltiMate 3000 RSLC nano System (Thermo Fisher Scientific) interfaced via a Nanospray Flex nanoelectrospray source (1). Briefly, reverse phase peptide separation was accomplished using a trap column (300 μm ID × 5 mm) packed with 5 μm 100 Å PepMap C18 medium coupled to a 50-cm long × 75 µm inner diameter analytical column packed with 2 µm 100 Å PepMap C18 silica (Thermo Fisher Scientific). Column temperature maintained at 50°C. Mobile phase solvent A was 2% acetonitrile (ACN), 98% water and 0.1% Formic Acid (FA). Mobile phase solvent B was 80% ACN, 20% water and 0.1% FA. Samples in loading buffer (3% ACN, 0.1% FA) loaded to column at a flow rate of 5.0 µl/min for 5 min, and eluted at a flow rate of 200 nl/min, using a 160-min LC gradient: 6.5 to 27% of solvent B in 110 min, 27-40% of B in next 15 min, 40-100% of B in next 10 min at which point the gradient was held at 100% of B for 10 min before reverting back to 2% of B, and hold at 2% of B for next 15 min for column equilibration. The column was further washed and equilibrated using three 30-min LC gradient, before injecting the next sample. All data acquired in the Orbitrap mass analyzer at resolution of 120,000 for MS1 and 15,000 for MS/MS at 200 m/z.

**Mass Spectrometry Data Analysis:** LC-MS/MS data were analyzed using MaxQuant software (version 1.6.3.3) against the combined non-redundant human protein sequence database from UniProt, for protein identification and label-free quantitation. The following parameters were used for database searches: precursor mass tolerance of 10 ppm; enzyme specificity of trypsin/Lys-C enzyme allowing up to 2 missed cleavages; oxidation of methionine (M) as a variable modification and iodoethanol (C) as a fixed modification. False discovery rate (FDR) of peptide spectral match (PSM) and protein identification was set to 0.01. Proteins with LFQ # 0 and MS/MS (spectral counts) ≥ 2 considered as identified and used for statistical analysis and visualization in Perseus platform (2). Statistical analysis performed using Analysis of Variance (ANOVA) and two-tailed Student’s t-test. Proteins with a p value ≤ 0.05 and absolute Log2 (LFQ) ≥ 0.50 were considered significantly regulated.

**HBV Infection:** HepG2-NTCP cells seeded onto collagen-coated 24-well plates, with density of 2×10^5^ cells/well, were grown in regular DMEM/F12 medium supplemented with 10% FBS, 100 U/ml penicillin, and 100 μg/ml streptomycin overnight. Next, they were switched to DMEM, 10% FBS, 2% DMSO, 100 U/ml penicillin, and 100 μg/ml streptomycin. After 24 h of 2% DMSO treatment, cells were infected with HBV virus at 500 vge/cell. The viral stock was diluted in DMEM supplemented 2% DMSO, and 4% PEG. The inoculum volume was 200 μl /plate; cells were then spinoculated for 1h at 1,000 × g at 37 °C. After 24h, inoculum was removed, cells were washed five times with PBS and maintained in DMEM supplemented with 10% FBS and 2% DMSO before harvest.

**Flow cytometry for quantification of HBV infected cells:** HBV infected HepG2-NTCP cells were trypsinized and washed with Phosphate buffered saline (PBS) on day 8 post-infection, and pelleted at 2500 rpm for 5 min. Subsequently, cells were stained with eBioscience Fixable viability dye eFluor 780, diluted in PBS (1:2000), and incubated on ice for 30 min, followed by washing with PBS. Cells were incubated with fixation and permeabilization buffer for 30 min at room temperature and washed with 1x permeabilization buffer. Intracellular staining was carried out by incubating cells with primary HBc Ab (1:10000) or IFI16 Ab (1:1000) (Santa-Cruz) in permeabilization buffer for 40 min at room temperature. Cells were washed twice with permeabilization buffer, incubated with the cocktail of Alexa-546 goat anti-rabbit Ab and Alexa-647 goat anti mouse IgG (Invitrogen A21235) for 40 min at room temperature. Cells were washed with permeabilization buffer twice, and once with PBS and permeabilization (1:1), fixed with 2% formaldehyde for 20 mins, washed with permeabilization buffer and analyzed by flow cytometry in an Attune NxT Flow Cytometer (Thermo Fisher). Data analysis performed using FlowJo software 10.8.1.

**Super Resolution Microscopy:**

**Fixation of Cells:** HepaRG cells were seeded on high-precision coverslips in 6-well culture plate and treated with 500 ng/ml IFN-α for 24 h. Cells were washed with 1x PBS followed by fixation with 4% paraformaldehyde for 15 min. Permeabilization was accomplished with 0.5% triton x-100 in 1xPBS for 10 min followed by blocking for 1 h with 5% normal goat serum and 0.3% triton x-100 in 1xPBS. After blocking, cells were incubated at 4 °C overnight with anti-DDX5 primary antibody (67025-1-IG, Proteintech, 1:400 dilution) and anti-IFI16 primary antibody (ab169788, Abcam, 1:100 dilution). Cells were washed three times for 5 min each time in 0.05% Triton X-100 in 1× PBS, and incubated at room temperature for 1 h with goat anti-mouse IgG (H+L) Cross-Adsorbed Secondary Antibody, Alexa Fluor™ 647 (A-21235, Invitrogen at 1:400 dilution), and goat anti-rabbit IgG (H+L), highly cross-adsorbed, CF™660C antibody (SAB4600453, Sigma, 1:400 dilution). After being washed three times for 5 min each time in 0.05% Triton X-100 in 1× PBS, cells were stored in 1× PBS at 4 °C.

**Imaging buffers and sample mounting:** The imaging buffer was prepared freshly, using 10 mM 2-mercaptoethylamine, 50 mM 2-mercaptoethanol, 2 mM cyclooctatetraene, 2.5 mM protocatechuic acid and 50 nM protocatechuic dioxygenase added in dSTORM base buffer containing 50 mM Tris, pH 8.0, 50 mM NaCl and 10% glucose, pH=8.0 (3). To mount the sample for imaging, a coverslip was placed with the sample on a custom-made metal holder. Subsequently, the imaging buffer was added on top of the coverslip and then placed another cleaned coverslip on it. To prevent oxygen exposure, the coverslip sandwich was sealed using a two-component silicone dental glue.

**Microscope setup:** Two-color super-resolution data were collected using a custom-designed single molecule localization microscopy (SMLM) setup with an Olympus IX-73 microscope stand. This system incorporates a 100x/1.35-NA silicone oil-immersion object lens (UPLSAPO100XS, Olympus America), a PIFOC objective positioner (ND72Z2LAQ, Physik Instrumente), and a three-axis piezo nano-positioning system. A laser line at wavelengths of 642 nm (2RU-VFL-P-2000-642-B1R, MPB Communications) was coupled into a polarization-maintaining single-mode fiber and an acousto-optic tunable filter (AOTFnC-400.650-TN) was used to manipulate excitation intensity. Emission spectra were split into conventional fluorescence and salvaged fluorescent by a dichroic mirror (ZT405/488/561/647rpc, Chroma). We applied an emission filter (FF01-731/137-25, Semrock) before collecting conventional fluorescence channel and another emission filter (FF01-661/20-25, Semrock) for salvaged fluorescence channel.

**SMLM acquisition and reconstruction:** Fluorescent-labeled samples were imaged at a laser intensity of approximately 5.3 kW/cm^2^ with a frame rate of 50 Hz. 40,000 frames were recorded for each region of interest. Image reconstruction and color assignment followed previously described methods (4, 5). Briefly, we reconstructed the conventional fluorescence data collected by the main sCMOS camera based on maximum likelihood estimation using 2D Gaussian methods within the situ point spread function retrieval (INSPR) toolbox (6). Each localization was then assigned to either Alexa Flour 647 or CF660C based on the intensity ratio between conventional fluorescence channel and salvaged fluorescence channel. We manually determined the threshold for rejecting localizations that deviated from the intensity ratio distribution to minimize cross-talk between two colors. When calculating RE of cropped sub-regions, a circular mask slightly smaller than the analyzed area was applied to the primary species to compensate for edge effects.

**Simulation of two molecular species testing relative enrichment method**

To verify the performance of relative enrichment method, we simulated two uniformly distributed molecular species within a 128$\times$128 pixels (12 nm pixel size) area using MATLAB. We simulated 1000 and 5000 molecules of each species to represent sparse and dense conditions, respectively. The results demonstrate that, in both conditions, the relative enrichment value approaches 1, as expected for two uniformly distributed species. Additionally, in another scenario where one species is consistently within 10 pixels of another uniformly distributed species, the relative enrichment value is greater than 1, indicating increased molecular co-localization.

Due to the stochastic blinking nature of the probes used in single-molecule localization microscopy, repeated localizations of a single molecule are commonly observed in the results. To test the robustness of the relative enrichment method in the presence of repeated localizations, we introduce a random number of repeated localizations for each simulated molecule. The position of these repeated localizations follow a Gaussian distribution, centered at the original molecule’s position, with a standard deviation equal to the localization precision, which is set to 10 nm. The results showed that the relative enrichment values remained consistent, regardless of the presence of repeated localizations.

**References**

1. Barabas AJ, Aryal UK, Gaskill BN. Proteome characterization of used nesting material and potential protein sources from group housed male mice, Mus musculus. Sci Rep 2019;9:17524.

2. Tyanova S, Temu T, Sinitcyn P, Carlson A, Hein MY, Geiger T, Mann M, et al. The Perseus computational platform for comprehensive analysis of (prote)omics data. Nat Methods 2016;13:731-740.

3. Olivier, N., Keller, D., Gönczy, P. & Manley, S. Resolution Doubling in 3D-STORM Imaging through Improved Buffers. *PLOS ONE* **8**, e69004, doi:10.1371/journal.pone.0069004 (2013).

4. Huang, F. *et al.* Video-rate nanoscopy using sCMOS camera–specific single-molecule localization algorithms. *Nature Methods* **10**, 653-658, doi:10.1038/nmeth.2488 (2013).

5. Zhang, Y. *et al.* Nanoscale subcellular architecture revealed by multicolor three-dimensional salvaged fluorescence imaging. *Nature Methods* **17**, 225-231, doi:10.1038/s41592-019-0676-4 (2020).

6. Xu, F. *et al.* Three-dimensional nanoscopy of whole cells and tissues with in situ point spread function retrieval. *Nature Methods* **17**, 531-540, doi:10.1038/s41592-020-0816-x (2020).

**Supporting Table S1: List of Plasmids, siRNAs**

| **Plasmids, siRNAs** | **Source** |
| --- | --- |
| prcccDNA | Generous gift from Prof. Dr. Qiang Deng |
| pCMV-Cre | Generous gift from Prof. Dr. Qiang Deng |
| pCAG-GFP-Cre | Addgene (#13776) |
| pCMV-FLAG-IFI16 | Addgene (#35064) |
| siCtrl | ThermoFisher Scientific (#4390843) |
| siDDX5 | ThermoFisher Scientific (#s4007) |
| siSUZ12 | ThermoFisher Scientific (#140248) |
| siEZH2 | ThermoFisher Scientific (#107417) |
| siRBBP4 | ThermoFisher Scientific (#s11838) |

**Supporting Table S2: Antibodies**

| **Antibody** | **Dilution** | **Application** | **Source** |
| --- | --- | --- | --- |
| Rabbit HBV HBc/ Core Antigen | 1:5000 in 2% BSA in TBST/1:2000 in 2% BSA  1:10,000 in Perm buffer | Western blot  Immunofluorescence  Flow cytometry | Dr. Adam Zlotnick |
| IFI16 | 1:1000 in 2% BSA  1:500 in Perm buffer | Western Blot  Flow cytometry | Santa Cruz |
| FLAG M2 | 1:2000 in 2% BSA TBST/1:1000 in 2% BSA  1:1000 | Western blot,  Immunofluorescence  Chromatin immunoprecipitation (ChIP) | Sigma (#F1804) |
| Mouse Human Actin | 1:1000 in 2% BSA in TBST | Western Blot | Sigma (#A5441) |
| Rabbit IgG | 5 μ*g* | Immunoprecipitation | Millipore Sigma (#17-700) |
| Mouse IgG | 5 μ*g* | Immunoprecipitation | Millipore Sigma (#17-700) |
| DYKDDDDK Magnetic agarose beads |  | Immunoprecipitation | ThermoFisher Scientific (#A36797) |
| Anti-acetyl-Histone H3 | 5 μg | ChIP | Sigma Aldrich (#06-599) |
| H3K27me3 | 5 μg | ChIP | Abcam (#ab6002) |
| FLAG | 5 μg | ChIP | Cell Signaling Technology (#14793S) |
| IFI16 | 5 μg | ChIP | ThermoFisher (#PA5-76462) |
| RBBP4 | 1:1000 in 2% BSA in TBST | Western Blot | ThermoFisher (#20364-1-AP) |
| EZH2 | 1:1000 in 2% BSA in TBST | Western Blot | Cell Signaling Technology (#3147S) |
| SUZ12 | 1:1000 in 2% BSA in TBST | Western Blot | Abcam (#ab126577) |
| DDX5 | 1:1000 in 2% BSA in TBST | Western Blot | Cell Signaling Technology (#9877S) |

**Supporting Table S3: Primer and RNA oligonucleotide sequences**

| **Primer** | **5’ – Sequence – 3’** |
| --- | --- |
| GAPDH-F | CCCTTCATTGACCTCAACTACA |
| GAPDH-R | ATGACAAGCTTCCCGTTCTC |
| Actin-F | GGCATGGGTCAGAAGGATT |
| Actin-R | GGGGTGTTGAAGGTCTCAAA |
| UBc-F | CCTGGAGGAGAAGAGGAAAGAGA |
| UBc-R | TTGAGGACCTCTGTGTATTTGTCA |
| HBV total RNA-F | TCACCAGCACCATGCAAC |
| HBV total RNA-R | AAGCCACCCAAGGCACAG |
| HBV preC/pgRNA-F | GAGTGTGGATTCGCACTCC |
| HBV preC/pgRNA-R | GAGGCGAGGGAGTTCTTCT |
| IFI16-F | ACTGAGTACAACAAAGCCATTTGA |
| IFI16-R | TTGTGACATTGTCCTGTCCCCAC |
| HBV rcccDNA-F | GTATTTCCCTGCTGGTGGC |
| HBV rcccDNA-R | GGTGAGTGATTGGAGGTTG |
| HBV cccDNA-F | CTCCCCGTCTGTGCCTTCT |
| HBV cccDNA-R | GCCCCAAAGCCACCCAAG |
| β-Globin-F | AGGTACGGCTGTCATCACTTAGA |
| β-Globin-R | CATGGTGTCTGTTTGAGGTTGCTA |

**Supporting Table S4: Reagents, Chemical inhibitors, and Kits**

| **Reagents, Chemical inhibitors, Kits** | **Source** |
| --- | --- |
| IFN-α | Millipore Sigma (#SRP4596) |
| PEG8000 | Sigma (#P5413) |
| Hoerchst 33342 | ThermoFisher Scientific (#62249) |
| PCR Mycoplasma Detection Kit | Abcam (#G238) |
| Cell Lysis Buffer (10X) | Cell Signaling Technology (#9803) |
| LightCycler® 480 SYBR Green I Master | Roche (#04887352001) |
| iScript™ cDNA Synthesis Kit | Biorad (#1708891) |
| Nitrocellulose Membrane, Roll, 0.2 µm | Biorad (#1620112) |
| LightCycler® 480 Sealing Foil | Roche (#04729757001) |
| LightCycler® 8-Tube Strips (white) | Roche (#06612601001) |
| DMSO | Sigma (#D8418-50ML) |
| Tween™ 20 | ThermoFisher Scientific (#BP337-500) |
| Tetracycline hydrochloride | Sigma (#T7660-5G) |
| Bovine Serum Albumin | Sigma (#A9647-100G) |
| Pierce™ ECL Western Blotting Substrate | ThermoFisher Scientific (#32106) |
| Pierce™ BCA Protein Assay Kit | ThermoFisher Scientific (#23227) |
| Lipofectamine™ 3000 Transfection Reagent | ThermoFisher Scientific (#L3000015) |
| Lipofectamine™ RNAiMAX Transfection Reagent | ThermoFisher Scientific (#13778150) |
| Restore™ PLUS Western Blot Stripping Buffer | ThermoFisher Scientific (#46430) |
| RNeasy Mini Kit | Qiagen (#74104) |
| Magna RIP™ RNA-Binding Protein Immunoprecipitation Kit | Millipore Sigma (#17-700) |
| Mercaptoethylamine | M6500, Sigma-Aldrich |
| cyclooctatetraene | 138924, Sigma-Aldrich |
| protocatechuic acid | 37580, Sigma-Aldrich |
| protocatechuic dioxygenase | P8279, Sigma-Aldrich |
| PrimeScript™ RT Reagent Kit with gDNA Eraser (Perfect Real Time) | 638951, TAKARA |
